# Bimetallic (Fe–Ga) Metal–Organic Frameworks for Tailoring Peroxidase-Like Activity: An Approach for Methane Partial Oxidation

**DOI:** 10.1021/acsmaterialsau.5c00045

**Published:** 2025-07-02

**Authors:** Gustavo Felix Bitencourt, Luana dos Santos Andrade, Wandson Lukas do Nascimento Amorim, Herich Henrique Lafayete Bastos Lima, Gabriela Tuono Martins Xavier, José Javier Sáez Acuña, Wagner Alves Carvalho, Mohamad El Roz, Thiago de Melo Lima, Dalmo Mandelli

**Affiliations:** † Centro de Ciências Naturais e Humanas, 74362 Universidade Federal do ABC—UFABC, Avenida dos Estados, 5001, Santo André/SP 09210-580, Brazil; ‡ Laboratoire Catalyse et Spectrochimie, University of Caen, UNICAEN, ENSICAEN, CNRS, Bd Maréchal Juin, 6, Caen/Calvados 14200, France; § Instituto de Química, 28110 Universidade Federal Fluminense—UFF, Outeiro de São João Batista, s/n, Niterói/RJ 24020-140, Brazil

**Keywords:** methane oxidation, bimetallic MOFs, heterogeneous catalysis, hydrogen peroxide, one-pot synthesis

## Abstract

Controllable methane oxidation directly into higher-value-added products under mild conditions remains a challenge due to the stability of the C–H bond. To promote methane oxidation using metal–organic frameworks, it is still necessary to explore ways of stabilizing metal active sites on MOFs due to the leaching and near-complete degradation of the catalyst after exposure to highly oxidative environments. Herein, we report a structural engineering approach based on Ga^3+^–Fe^3+^ complexes in biological systems to tailor the redox-cycle activity. It was imitated by tailoring Ga^3+^ doping into Fe-MIL-88B. Thus, novel MOFs with differing compositions of Fe and Ga were synthesized and denoted as Fe_*x*_Ga_*y*_-MOF. Chemical stability tests in water and oxidative environments confirmed that the bimetallic MOFs indeed exhibited higher stability with reduced leaching of iron sites. Fe_0.3_Ga_0.7_-MOF was demonstrated to be the most stable material while being active and was selected for further catalytic evaluations. Several parameters for the methane oxidation reaction were optimized such as mass of catalyst, temperature, pressure, and others. Fe_0.3_Ga_0.7_-MOF exhibited a productivity of 29.9, 381.9, and 90.1 μmol g_cat_^–1^ for methanol, formic acid, and acetic acid, respectively. Compared to the Fe-MIL-88B, the Fe_0.3_Ga_0.7_-MOF had an enhancement of 36% toward the selectivity of oxygenates and also reduced by almost 95% the undesired evolution of CO_2_. This material demonstrated excellent stability, retaining its catalytic activity after three cycles with only 0.1% metal leaching, highlighting the effectiveness of the stabilization method. In contrast, Fe-MIL-88B showed poor stability, with 38.3% metal leaching after the first cycle. Mechanistic insights indicated a major role of reactive oxygen species in the formation of products.

## Graphical abstract

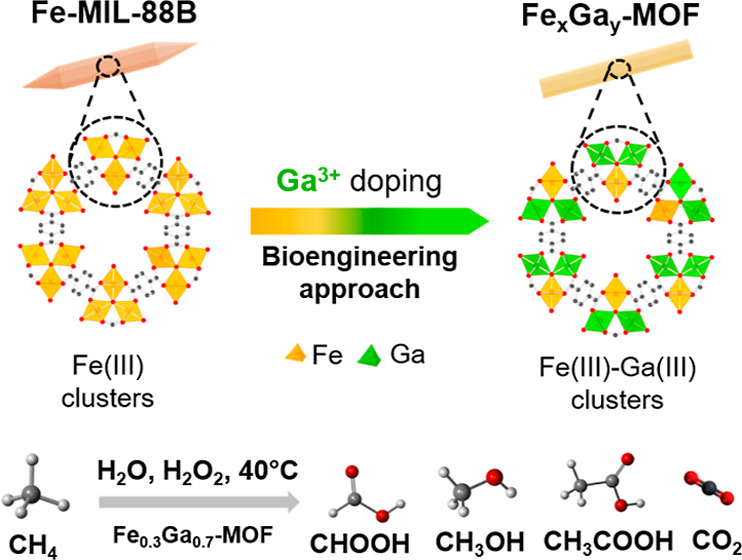

## 1 Introduction

Methane (CH_4_) is a potent greenhouse gas with a global warming potential that is over 25 times higher than that of carbon dioxide (CO_2_). It is a primary component of natural gas and is released during the production, transport, and use of fossil fuels. Methane emissions also occur naturally from wetlands, rice paddies, and the digestive processes of livestock.^1^ Additionally, it directly impacts local air pollution and public health, leading to respiratory problems and other health risks.^2^

One effective way to reduce methane emissions is to enhance methane partial oxidation, which is the process by which methane is converted into higher added-value products. Currently, traditional methane conversion primarily relies on indirect oxidation processes, characterized by high temperatures and pressures, typically involving methane reforming and Fischer–Tropsch synthesis. However, these methods are associated with costly and energy-intensive operations.^3^ Thus, there has been growing interest in converting methane directly into more valuable and useful chemicals, such as methanol, formic acid, and acetic acid, using mild conditions (low temperature, low pressure, and common oxidizing agents like H_2_O_2_ and/or O_2_).^4^ However, methane partial oxidation is a challenging process due to the high inertness of methane and the difficulty of activating its C–H bonds.^5^ In addition, there are several problems associated with the low reaction selectivity, toward partial oxidation versus total oxidation.^6^ This is due to the fact that the oxygenated molecules, produced during the reaction, are more reactive than methane, and undergoing overoxidation.^7^

Recent advances in catalysis have shown promising results in achieving this transformation. Approaches involve the use of copper- or iron-based catalysts that can activate methane through the hydrogen peroxide (H_2_O_2_) activation cycle and promote its conversion to oxygenates.^8,9^ For example, Zhao et al.^10^ demonstrated that iron oxides, such as Fe_2_O_3_, exhibit activity toward methane oxidation; however, they reported a relatively low productivity of 258 μmol g_cat_^–1^ alongside the formation of 92 μmol g_cat_^–1^ of CO_2_. In contrast, FeO selectively converts methane to CO_2_, which is an undesirable pathway. In light of these challenges, metal–organic frameworks (MOFs) have garnered significant attention in recent years due to their unique structural and chemical properties, which may provide enhanced catalytic performance compared with traditional metal oxides. Their tunable porosity and the ability to incorporate various metal centers make MOFs highly attractive for a wide range of applications, particularly as potential catalysts for alkane oxidation.^11^ In 2018, Osadchii et al.^12^ employed a postsynthetic modification and electrochemical methods to introduce iron sites into the Al-MIL-53, resulting in a stable support for methane oxidation. Although they achieved notable results, such as 77% selectivity toward oxygenates and a significant reduction of CO_2_ evolution to 23%, the main product that was methanol accounted for only a maximum distribution of 51.6% among the other oxygenates, which presents a significant limitation. Similarly, in 2022, Lee et al.^13^ developed a material in which the active phase comprised Cu­(II) species doped into the ZIF-7 structure. This material demonstrated a production of 612.7 μmol g_cat_^–1^ of oxygenates (15%), yet it also exhibited a substantial CO_2_ productivity of 3498 μmol g_cat_^–1^ (85%), attributed to ligand oxidation processes. While the productivity metrics are impressive, the multistep synthesis involved in catalyst preparation remains a considerable drawback.

In this scenario, Fe-MIL-88B present a good candidate for this reaction due to the flexibility of its three-dimensional structure, and they are very organized trimeric iron­(III) nodes, facilitating Fenton-like reactions.^14^ However, a challenge associated with some MOFs is their tendency to undergo structural changes or collapse under certain conditions.^15^ Factors such as moisture, oxidative, or acidic environments can lead to loss of porosity, crystallinity, and overall performance.^16^ This underscores the importance of assessing the MOF stability to ensure long-term functionality. It also highlights the need for innovative stabilization strategies, such as metal doping, core–shell techniques, mixed linker modulation, and functionalization with biomolecules, to enhance MOF durability in challenging environments.^17–21^

Among these strategies, mixed-metal or bimetallic MOFs have gained attention as an effective approach to improve both stability and catalytic performance. By incorporation of two different metal ions within the framework, these materials benefit from synergistic electronic and structural effects, which can enhance metal–ligand interactions and mitigate common issues such as metal leaching or framework collapse. Depending on the metal distribution and incorporation, bimetallic MOFs can adopt either a homogeneous structure, where both metals are uniformly integrated into the same nodes, or heterogeneous configurations, where metals occupy distinct positions within the framework and even led to amorphous structures.^22^ These materials have demonstrated superior properties in catalysis, optoelectronics, and energy storage due to their tunable electronic environments and improved charge transfer.^23,24^

Building on these principles, this work focuses on the development of bimetallic MOFs (Fe_*x*_Ga_*y*_-MOFs) through a one-pot synthesis method, offering a simple alternative to the more complex, multistep postsynthetic modification processes. Our focus centers on addressing challenges in catalysis and material stability, particularly through the structural engineering of Fe-MIL-88B for oxidative processes. By doping Fe-MIL-88B with Ga^3+^, we aim to replicate the metabolic processes of the bacterium *Pseudomonas aeruginosa*,^25^ whose antioxidant mechanism relies on Fe^3+^ coordination at specific active sites to convert H_2_O_2_ into active radicals. In the presence of Ga^3+^, these ions mimic Fe^3+^ coordination, thereby modulating the redox cycle activity in the cells and reducing the H_2_O_2_ consumption.^26^ Furthermore, the similar ionic radii and charge (3+) of gallium­(III) and iron­(III) ensure a favorable phase match during MOF synthesis.^26^ Thus, taking into consideration these properties, other metals in the same period of iron in the periodic table were not considered for this study. To assess the material’s performance, we conducted chemical stability tests in water and oxidative environments, evaluating the potential for enhanced stability in catalytic applications such as methane partial oxidation. Optimization of various reaction parameters led to the development of a stable and recyclable catalyst for methane partial oxidation in mild conditions using H_2_O_2_ as the oxidant.

## 2 Experimental Section

### 2.1 Materials

Iron­(III) chloride hexahydrate (FeCl_3_·6H_2_O, 98%), gallium­(III) nitrate hydrate (Ga­(NO_3_)_3_·*x*H_2_O, 99.9%), terephthalic acid, and *N*,*N*-dimethylformamide (DMF, 99%) were purchased from Sigma-Aldrich. Sulfuric acid (H_2_SO_4_), hydrochloric acid (HCl), hydrogen peroxide (H_2_O_2_), and sodium hydroxide (NaOH) were purchased from Synth (Brazil). All reagents were used without further purification.

### 2.2 Synthesis of MOFs

The pristine Fe-MIL-88B MOF was synthesized following a method described in the literature, with modifications.^27,28^ The synthesis involved the use of 10 mmol of FeCl_3_·6H_2_O, 10 mmol of terephthalic acid, 8 mL of 1 mol L^–1^ NaOH, and 50 mL of DMF as reagents. To synthesize Ga-MIL-53, the same method as that for Fe-MIL-88B was employed, but 10 mmol of Ga­(NO_3_)_3_·*x*H_2_O was used as the precursor salt. Additionally, different ratios of metal precursor were used in the synthesis, leading to different Fe_*x*_Ga_*y*_-MOF samples with various Fe/Ga ratios. For example, the Fe_0.3_Ga_0.7_-MOF was obtained by dissolving FeCl_3_·6H_2_O (3 mmol) and Ga­(NO_3_)_3_·*x*H_2_O (7 mmol) in 10 mL of DMF. The Fe^3+^ and Ga^3+^ solution was introduced into a Schott flask and stirred for 20 min, followed by the addition of the ligand solution (10 mmol) along with 20 mL of DMF. Subsequently, 8 mL of 1 mol L^–1^ NaOH (in water) was added dropwise to the flask while stirring. After 40 min, the system was incubated in an oven at 100 °C for 12 h. After cooling for 1 h, the resulting MOF was washed with DMF, ethanol, and deionized water, until the supernatant became colorless. The MOF was then collected through centrifugation, dried overnight at 110 °C, and vacuum-activated at 150 °C for 12 h. The bimetallic MOFs will be denoted in the manuscript as Fe_0.7_Ga_0.3_-MOF, Fe_0.5_Ga_0.5_-MOF and Fe_0.3_Ga_0.7_-MOF, which is based on the synthesis feeding molar ratio.

### 2.3 Characterization

The powder X-ray diffraction (XRD) data were obtained using Bruker AXS-D8 Focus with Cu Kα (λ = 1.5606 Å) radiation source, scanning between 2θ range of 4–55° at a rate of 0.02 min^–1^.

Fourier transform infrared spectroscopy (FT-IR) was performed using a Varian Agilent 640 spectrometer with direct sample insertion into the ATR. The spectrum was measured ranging between 4000 cm^–1^ and 500 cm^–1^ with a resolution of 16 scans.

Raman spectroscopy was performed on a micro-Raman setup (Una LabRam HR800 Jobin Yvon spectrometer). A laser with a 532 nm wavelength was used as the excitation source, with a maximum power of 1 mW.

Nitrogen, carbon, and hydrogen contents were studied by the elemental analyzer FlashEA 1112 from Thermo Scientific. The samples were degassed overnight before analysis, and duplicates of each sample were prepared. The oxygen content was determined by difference, considering all inorganic compositions (Fe or Ga) in addition to C, N, and H.

Textural property characterization was performed via N_2_ physisorption at 77 K using the Quantachrome Autosorb 1 Surface Area and Pore Size Analyzer. Samples were activated before analysis under vacuum at 150 °C for 24 h. The surface area was obtained by the Brunauer–Emmett–Teller method (BET) in the *P*/*P*_0_ range = 0.05–0.30, total pore volume was calculated at *P*/*P*_0_ = 0.97, average pore diameters were obtained by the Barrett–Joyner–Halenda (BJH) method, and the density functional theory method was used to calculate the pore size distribution.^29^

Micrographs of the materials were obtained using an FEI Quanta 250 Scanning Electron Microscope, while the elemental mapping by SEM–EDS was obtained in a Compact SEM JEOL/EO. For sample preparation, materials were dispersed in deionized water and sonicated for 5 min in an ultrasonic bath. Then, 3 μL of the sample was dispersed over carbon tape on the stub. After drying, the samples were coated with 20 nm of Au using sputtering equipment (Leica ACE200).

Regarding transmission electron microscopy, a high-resolution transmission electron microscope (Thermo Fischer Talos F200X-G2) was operated at a 200 kV accelerating voltage using a field-emission gun. The diffraction patterns were obtained by the selected-area electron diffraction (SAED) technique using a physical aperture of 900 nm.

Thermogravimetric analysis was conducted using a TGA/DSC 1 STAR^e^ equipment in an air atmosphere. For these analyses, 2 to 4 mg of each sample was weighed in an alumina crucible. The analysis parameters were a heating rate at 5 °C min^–1^, under a flow rate of 50 mL min^–1^ of gas until a temperature of 600 °C was reached.

The inorganic composition of each catalyst was obtained using inductively coupled plasma optical emission spectrometry equipment iCap7600 (ICP OES). Solid samples were digested in a microwave (Biotage initiator+) and then filtered with 0.45 μm PVDF syringe filters. The digested samples were diluted according to the calibration curves of Fe^3+^ and Ga^3+^. The method of digestion involved adding, in a 10 mL capped microwave vial, 2.5 mL of HNO_3_, 0.62 mL of 30% H_2_O_2_ 30%, 0.31 mL of HCl, and 2–3 mg of the sample. The samples were subjected to microwave irradiation (150 W) at 180 °C for 10 min until the solution became completely clear.^29^ Quantification of Fe^3+^ and Ga^3+^ in liquid phase samples (stability tests, catalytic reactions, and leaching tests) were analyzed by flame atomic absorption spectrometry (FAAS, Contra300, Analytik Jena AG).

X-ray photoelectron spectroscopy (XPS, Thermo Scientific ESCALAB 250Xi) was used to investigate the chemical composition of the materials. The spectra were acquired using an Al Kα radiation source (1486.6 eV), and all spectra were calibrated using an adventitious C 1s peak centered at a 284.8 eV binding energy.

### 2.4 Chemical Stability Tests in Aqueous and Oxidative Environments

The MOFs were subjected to two different conditions: immersion in water for 7 days and exposure to water containing hydrogen peroxide (H_2_O_2_) at a concentration of 0.65 mol L^–1^ for 24 h at room temperature (25 °C ± 1). Water supernatant aliquots were subsequently analyzed by using flame atomic absorption spectroscopy (FAAS) to determine the leaching of Fe^3+^ and Ga^3+^ ions in the solution. The quantification was made in triplicate and the value was expressed as an average of the values (error <0.04%). In parallel, the remaining MOF samples were characterized to assess differences before and after the stability tests. Scheme S1 illustrates the generation of both liquid-phase and solid-phase samples in each experiment.

### 2.5 Catalytic Experiments

The methane oxidation reaction was carried out in a Teflon-lined stainless-steel autoclave, as illustrated in Figure S2a. Different amounts of MOF, deionized water (solvent), H_2_O_2_ (oxidizing agent), and CH_4_ (substrate) were added to the reactor. The autoclave was sealed, and methane was introduced until the desired pressure was achieved. The solution within the autoclave was stirred at different temperatures from 5 min to 5 h. Upon completion of the reaction time, the autoclave was placed in an ice bath for 5 min to prevent volatilization of any desired product. Then, the pressure of the system was released, and a 100 μL aliquot of the prefiltered reaction mixture was collected. This sample was diluted in a vial containing 400 μL of deionized water, and then triphenylphosphine (PPh_3_) was added to convert possible intermediate species such as methyl hydroperoxide (CH_3_OOH) to the alcohol form (CH_3_OH).^30^ Then, the quantification of liquid phase products in this aliquot was performed by GC–MS (SHIMADZU QP-2010Plus). Some other experiments, such as blank tests, were carried out and will be further discussed.

The CO_2_ produced in the reaction was quantified by GC–MS in SIM mode, using the Ar/N_2_ ratio presented inside the reactor as an internal standard. Calibration curves from 0 to 5 bar of CO_2_, maintaining a total pressure of 20.7 bar of CH_4_, were made. The *m*/*z* used in the mass spectrometer were 40 *m*/*z*, 28 *m*/*z*, and 44 *m*/*z* for Ar, N_2_, and CO_2_ respectively. The gas samples were collected using a system specially developed in the laboratory for this purpose (Figure S2b).

The hot filtration test (Sheldon’s test) was conducted to determine the heterogeneous nature of the catalytic reactions.^31^ As can be seen in Scheme S2, this test consists of the filtration of the catalyst and evaluates the progress or not in productivity concerning the presence and absence of catalytic species. The heterogeneous nature of catalysis can be confirmed if the productivity does not increase due to the absence of active sites for the reaction. However, if productivity still rises, it can be inferred that active sites leach from the catalyst, and the reaction occurs between the dissolved metallic species and the substrate, indicating a homogeneous-based catalysis.

The recycling tests were performed according to Scheme S3. The reactions were made at 40 °C with 20.7 bar CH_4_, 20 mg of MOF, 1000 rpm, 2 mL of H_2_O, and 50 μL of 60% H_2_O_2_ (1.31 mmol) for 1 h. At the end of each reaction cycle, the catalyst was recovered from the reactor, centrifuged, and dried at 125 °C overnight. Then, each MOF went through the same reaction conditions for three consecutive cycles.

The remaining H_2_O_2_ concentration in the reaction system was quantified by iodometry.^32^

The experiment of O_2_ evolution by the H_2_O_2_ decomposition was conducted in a round-bottom flask with two necks, directly connected to a condenser, where the cooling water was maintained at 15 °C. The condenser gas outlet was connected to an apparatus for volumetric quantification of the gas released during the experiment (Figure S3).^33^

Aiming to evaluate the regioselectivity and link it to the reaction mechanism, the *n*-heptane oxidation reactions were made in the same autoclave as the methane oxidation, and the products were quantified by GC–MS. The reaction conditions were similar to the methane oxidation for the sake of comparison. The solvent was switched to acetonitrile because of the high insolubility and heterogeneity of *n*-heptane in water (Scheme S4).

## 3 Results and Discussion

### 3.1 MOFs Characterization

Figure 1a shows the one-pot synthesis scheme used to obtain the novel bimetallic MOFs with different ratios of Ga^3+^ doping in the pristine MOF, exposing mixed Ga–O–Fe nodes and also their powder aspect and color. The XRD pattern of the synthesized Fe-MIL-88B and Ga-MIL-53 were compared to the literature (Figures S5 and S6), confirming the achievement of the crystallographic pattern and validating the presence of the major crystalline peaks.^28,34^ It is important to highlight that because of the flexible nature of MIL-88B and MIL-53, some guest molecules of the synthesis like H_2_O or DMF can still be trapped inside of the framework and generate some shifts in the pattern.^34,35^ This is consistent with literature data that report that metal doping in the pristine MOF counterpart leads to a bimetallic MOF.^36–38^

**Figure 1:**
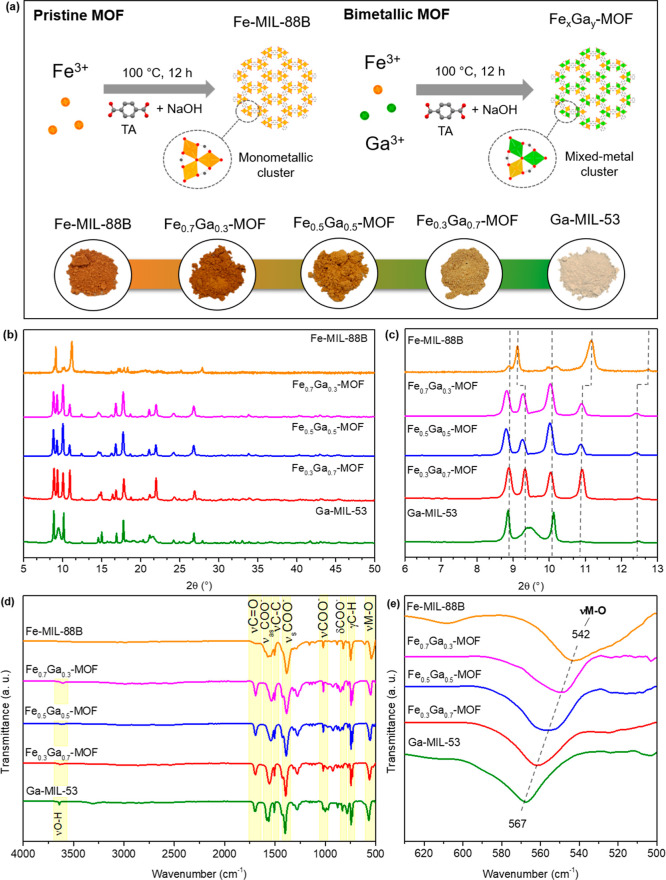
(a) Schematic synthetic route for comparing the pristine Fe-MIL-88B and the bimetallic Fe_*x*_Ga_*y*_-MOFs. The images show the powder aspect and color of the synthesized materials. TA = terephthalic acid. (b,c) Comparison of materials PXRD in different regions. (d) FT-IR ATR spectra of the synthesized MOFs. (e) Amplified region of FT-IR ATR spectra highlighting the M–O stretching.

Regarding the XRD comparison of the synthesized Fe_*x*_Ga_*y*_-MOFs (Figure 1b), it can be seen that the crystalline patterns slightly shifted and did not lead to amorphous phases when switching Fe^3+^ for Ga^3+^ in the structure,^22,39^ indicating that the new materials have a similar structure to the parents Fe-MIL-88B and Ga-MIL-53. For example, as seen in Figure 1c, the main diffraction peaks related to the Fe-MIL-88B structure are identified in 2θ = 9.09°, 9.98°, 11.04°, and 12.67°, which are correlated to the [002], [100], [101] and [102] crystal planes.^22^ Meanwhile, for the Fe_*x*_Ga_*y*_-MOF and Ga-MIL-53 these peaks are displaced to 8.79°/8.78°, 9.38°/9.34°, 9.96°/9.98°, and 12.38°/12.39°, respectively. The strong similarity in the crystallographic patterns indicates isomorphism across the bimetallic MOFs,^40^ while the minor differences can be attributed to the smaller ionic radius of Ga^3+^ compared to Fe^3+^. This suggests that the materials are solid solutions with a homogeneous distribution of bimetallic sites. Furthermore, compared to the parent Fe-MIL-88B and Ga-MIL-53, the bimetallic MOFs do not exhibit significant peak broadening or splitting, confirming that they consist of a single phase rather than a mixture of multiple phases.^40,41^

The FT-IR ATR spectra of the materials are shown in Figure 1d. All MOFs showed the same characteristic vibrational bands, with some minor deviation/shift, as all of them were made with the same organic linker (terephthalic acid). The spectra show a C=O stretch in 1693–1699 cm^–1^ related to the carboxylate group of the ligand.^27^ Then, different stretching of the carboxylate group (COO^–^) can be observed in 1550–1558 cm^–1^, 1380–1392 cm^–1^, and 1016–1020 cm^–1^, while in 827–810 cm^–1^ there is vibrational bending of COO^–^. The presence of double bands at 1507/1318–1324 cm^–1^ is related to the C–C ring stretching. Also, in 755–740 cm^–1^ the C–H bending is from the organic linker. In Figure 1e, the M–O stretching band (where M = Fe or Ga) is related to the oxo-clusters from 542 to 567 cm^–1^. It is worth noting that as Ga^3+^ is a smaller ion compared to Fe^3+^, this difference in size affects the bond strength between the metal and oxygen in the clusters. Typically, smaller cations form shorter and stronger M–O bonds, leading to a higher wavenumber in the IR spectrum,^42^ in agreement with our observation. Furthermore, the Raman spectra (Figure S6) of monometallic Fe-MIL-88B and Ga-MIL-53, compared with bimetallic Fe_0.3_Ga_0.7_-MOF, exhibit a similar pattern. Due to the same organic linker, all spectra appear similar; however, as the gallium content increases, the characteristic bands shift toward higher Raman wavenumbers. For example, the vibrational modes associated with metal–oxygen clusters (ν­(MO_2_C)) in the 1421–1458 cm^–1^ range demonstrate this trend. Fe-MIL-88B displays peaks at 1421 and 1452 cm^–1^, while Fe_0.3_Ga_0.7_-MOF exhibits a shift to 1435 and 1455 cm^–1^. The highest Raman shift is observed for Ga-MIL-53 at 1448 and 1458 cm^–1^.^12^ Thus, this progressive increase in the M–O stretching wavenumbers in FT-IR and overall band shifts in Raman with higher gallium incorporation provides further evidence of the effective integration of Fe–O–Ga nodes in the bimetallic MOF structure.^43^

According to Figure 2, Fe-MIL-88B shows a rod shape morphology, which is similar to other Fe-MIL-88B reported previously in the literature.^44^ It can be noted that all Fe_*x*_Ga_*y*_-MOFs have a rod-like shape, corroborating with the XRD and to the fact that incorporating gallium into the structure did not lead to amorphization or severe alteration in the morphology, as seen by other bimetallic MOFs.^22,45^ This small difference can be explained by the competition of Ga^3+^ and Fe^3+^ to coordinate with the organic ligand during the synthesis procedure, leading to a mismatch of metal–ligand and prioritizing the growth of the structure toward a specific crystal plane. Besides, Ga-MIL-53 morphology differs from the others, exposing small rounded aggregates with irregular lattices. All syntheses showed good reproducibility for homogeneous crystal size and morphology. Additionally, to guarantee that the bimetallic MOFs are not merely heterogeneous physical mixtures of Fe- and Ga-MOFs crystals, the SEM–EDS mapping (Figure 2) showed the presence of both metals very well dispersed and homogeneous in the isolated powder particles.^46^ In Figure S7a, a control experiment was made by preparing a physical mixture of Fe-MIL-88B and Ga-MIL-53 with an equimolar ratio to that of Fe_0.3_Ga_0.7_-MOF. The results show a nonmatching elemental mapping of Fe and Ga for the physical mixture and also the morphology change exposing rod particles with rounded aggregates attached, confirming the difference among the materials. The XRD of the Fe_0.3_Ga_0.7_-MOF and the physical mixture were also compared (Figure S7b,c), exposing the successful synthesis of the bimetallic MOFs in this work. The physical mixture shows exactly matching peaks with the pristine Fe-MIL-88B and Ga-MIL-53, indicating the two separate phases, while the Fe_0.3_Ga_0.7_-MOF did not. In Figure S7d, exposing the FT-IR ATR of this experiment also indicates a broadening in the M–O stretching of the physical mixture, separating between Ga–O and Fe–O contributions which are not from the same node—mixture of monometallic Fe–O–Fe and Ga–O–Ga clusters. Thus, all evidence confirms the single phase nature of Fe_*x*_Ga_*y*_-MOFs samples.

**Figure 2:**
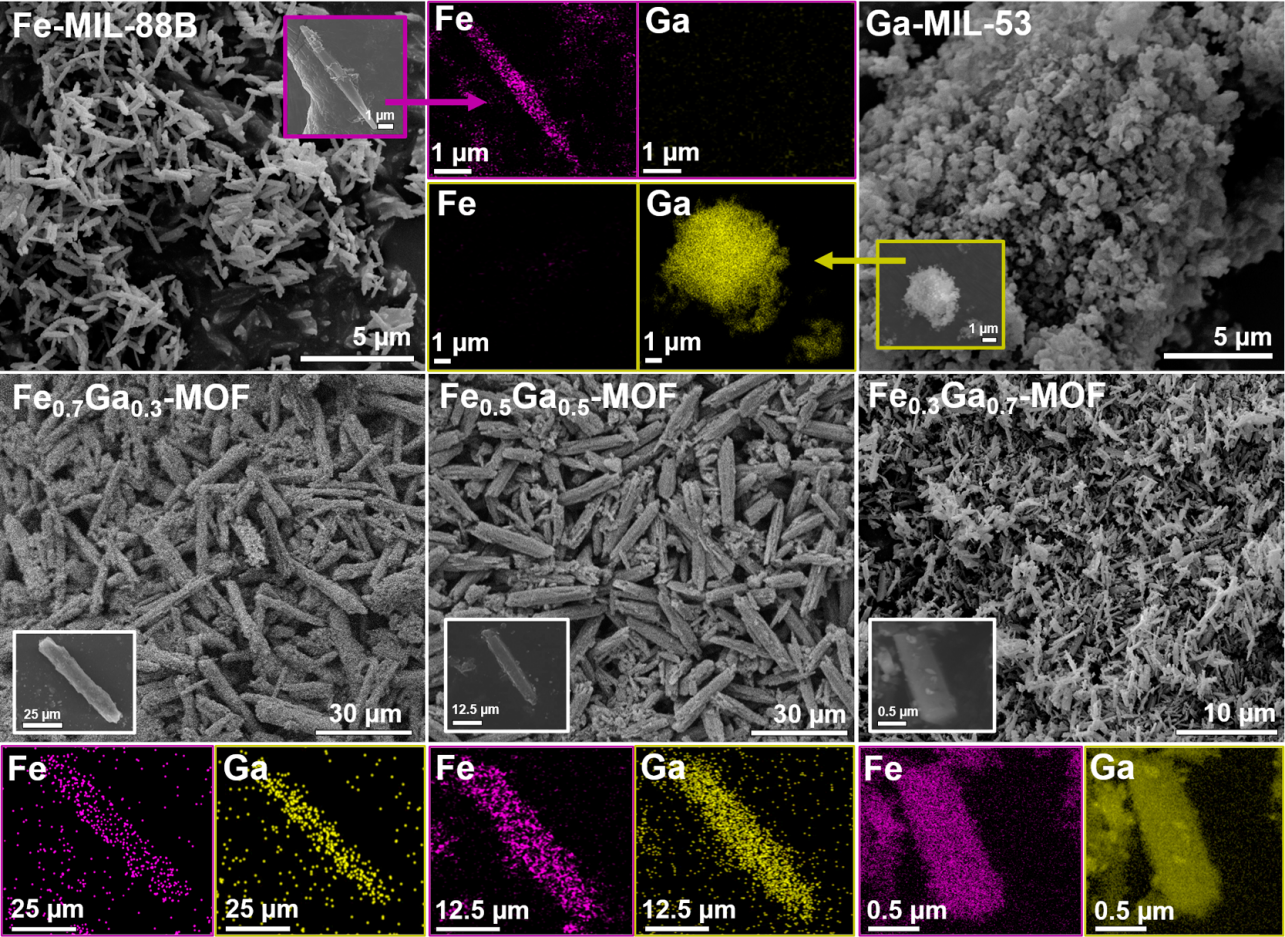
Comparison of the morphology of the MOFs by SEM and the elemental mapping (SEM–EDS) of Fe and Ga in an isolated powder particle.

To gain deeper insights into the structural characteristics of the bimetallic MOFs, transmission electron microscopy (TEM) analysis was performed on the Fe_0.3_Ga_0.7_-MOF. As observed in Figure 3a,b, the material predominantly exhibits a rod-like morphology. In addition to these larger particles (∼3.2 μm), smaller nanoparticles (∼174 nm) were also detected, adhering to the external surface of rod-like particles. This suggests the presence of a secondary growth phenomenon or nucleation effects during synthesis, potentially influenced by differences in metal (Fe and Ga) coordination dynamics and the used modulator.^22^ To further investigate the structural composition, high-resolution TEM (HRTEM) imaging was conducted. The analysis revealed regions exhibiting clear lattice fringes, indicative of the ordered crystalline structure (Figure 3c) alongside areas without discernible fringes (Figure 3d). The difficulty in identifying well-defined lattice structures across all regions implies that the MOF may be sensitive to the high-energy electron beam, leading to structural instability under prolonged beam exposure.^47,48^ Despite this challenge, through diffraction patterns, the measured lattice fringe spacings were determined to be 0.36, 0.28, and 0.22 nm, which correspond to the (321), (222), and (215) crystallographic planes of Ga-MIL-53 (CCDC no. 704888), respectively. These results reinforce the similar structure of the synthesized bimetallic MOFs compared to the pristine materials, being in agreement with our PXRD data (Figure 1) and also other mixed-metal based-MOFs in the literature.^49,50^ Furthermore, SAED patterns confirmed the polycrystalline nature of the material (diffraction rings; Figure 3e). To fully validate the compositional homogeneity of Fe_0.3_Ga_0.7_-MOF, high-angle annular dark-field scanning transmission electron microscopy (HAADF-STEM) was employed. As depicted in Figure 3f, EDS elemental mapping demonstrates a uniform distribution of Fe, Ga, C, and O throughout the structure. The presence of carbon and oxygen is attributed to the terephthalic acid linker, which coordinates with Fe and Ga to form metal clusters. Notably, even the smaller nanoparticles retained a similar elemental mapping, confirming their compositional consistency with that of the larger particles. A slight variation in density between the larger and smaller particles was observed, which may be attributed to subtle differences in the metal content between them. This variation likely arises from differences in the coordination kinetics of Fe^3+^ and Ga^3+^ alongside with the addition of NaOH during MOF synthesis, which accelerates the particle crystallization. Consequently, this could influence nucleation and growth processes, leading to the observed morphological disparities.^22,51^

**Figure 3:**
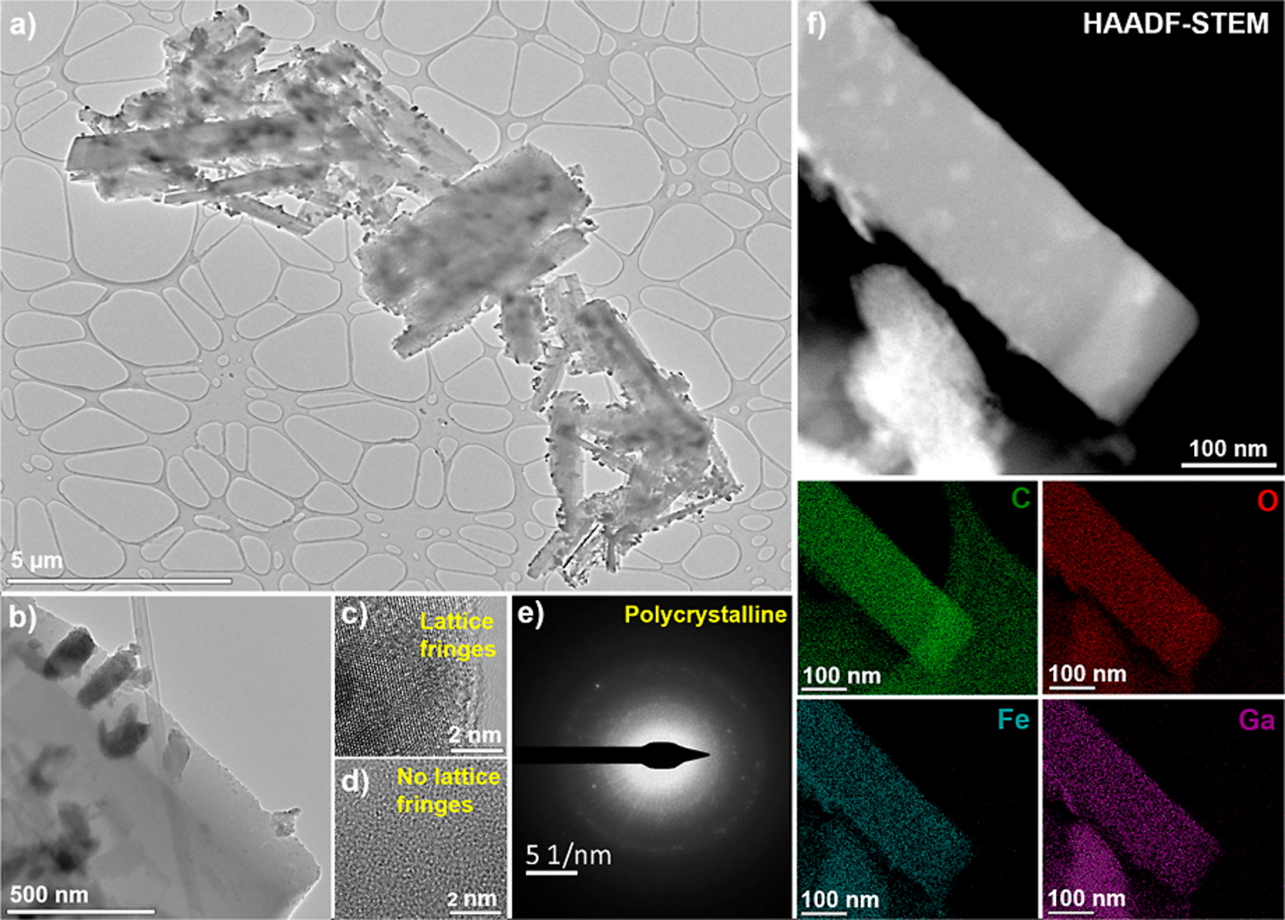
(a,b) TEM, (c,d) HRTEM, (e) SAED, and (f) HAADF-STEM of the Fe_0.3_Ga_0.7_-MOF.

Regarding the chemical composition, it can be seen in Figure 4a, in the full survey XPS spectra, that all materials have shown peak intensities similar to those of C and O, due to the presence of the same organic ligand. Simultaneously, the intensity of Fe and Ga varies as the metal doping ratio of Fe_*x*_Ga_*y*_-MOFs differs. Additionally, in XPS high-resolution spectra (Figures S8–S11), all materials showed similar deconvolution patterns of binding energy, indicating the successful synthesis of different bimetallic MOFs. For all materials, in C 1s spectra (Figure S8), deconvolution peaks near 284.8, 285.9, and 288.7 eV were attributed to the C–C, carboxylate (COO^–^), and C=O atoms of the terephthalic acid. In O 1s spectra (Figure S9), the region was deconvoluted into three peaks: 530.2, 531.9, and 533.8 eV, which were attributed to C–O–M (with M = Ga or Fe), M–O–M, and O–H bonds, respectively, from a typical MOF structure.^52^ In Fe 2p spectra (Figure S10 and Table S2), all Fe-based MOFs showed the same deconvolution of Fe into four peaks, which are the two satellite peaks near ∼729.3 and 715.4 eV, while showing the Fe 2p_1/2_ and Fe 2p_3/2_ near 724–725.2 eV and 710.8–711.8 eV, respectively, illustrating the presence of Fe^3+^ species. In the Ga 3d spectra (Figure S11 and Table S2), all Ga-based MOFs showed in 20.6–20.9 eV the main presence of Ga^3+^ species and/or Ga–O bonds, besides some minor peaks between 24.3 and 25.6 eV (peak fit 2) of O 2s overlap.^53,54^

**Figure 4:**
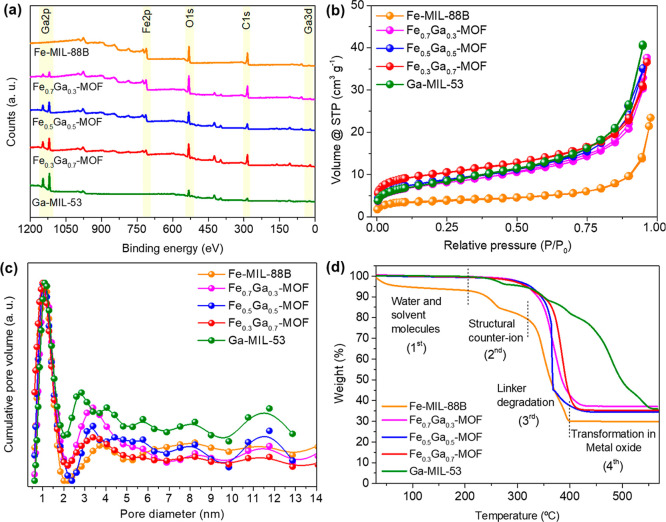
(a) Full survey XPS spectra of the MOFs. (b) N_2_-adsorption–desorption isotherms, (c) pore size distribution, and (d) thermogravimetric analysis among the MOFs.

The inorganic and organic composition of the synthesized materials (Table S3) was confirmed by ICP OES and CHNS analysis, demonstrating the successful modulation of iron and gallium contents within the MOF structure. For example, in Fe_0.7_Ga_0.3_-MOF, the synthesis molar feeding ratio was Fe/Ga = 70:30 (7 mmol:3 mmol, total 10 mmol), close to the experimental ratio (79:21). The slight difference observed between the theoretical and experimental molar ratios is probably attributed to the intrinsic properties of Fe^3+^ and Ga^3+^ and their differing coordination kinetics with terephthalic acid.^22^ Regarding the organic content derived from the ligand, CHNS analysis reveals that Fe-MIL-88B, Fe_*x*_Ga_*y*_-MOF, and Ga-MIL-53 exhibit very similar carbon, hydrogen, and oxygen contents, which are consistent with the terephthalic acid used in the synthesis. The minor variation of less than 2% further supports the successful synthesis, confirming that the primary structural difference lies in the metal nodes.^55^

Although MOFs are typically reported to have high surface areas,^56^ the case of MIL-88B is somewhat different due to its highly flexible structure. Simulations suggest that at full expansion (open form), MIL-88B could reach a surface area of 3040 m^2^ g^–1^.^57^ However, this flexibility, combined with a low affinity for N_2_ gas adsorption, results in inaccurately low surface area measurements. The structural dynamics and weak interaction with N_2_ limit the effectiveness of traditional surface area determination methods.^58^ The same flexible characteristic is also reported in the Ga-MIL-53.^34,59^ Thus, due to the similar properties between Fe­(III) and Ga­(III) octahedral complexes, like ionic radii which are 0.62 and 0.64 Å, these textural properties were also reproduced in the Fe_*x*_Ga_*y*_-MOFs, maintaining these characteristics. As shown in Table 1, all MOFs showed similar surface area (∼15–33 m^2^ g^–1^) and total pore volume (0.027–0.064 cm^3^ g^–1^). All samples presented a similar pore size distribution and a type-IV adsorption isotherm, implying multilayer physisorption and that all materials have mesoporous structures (Figure 4b,c).^60,61^

**Table 1:** Textural Analysis of the Synthesized MOFs^a^

sample	*S*_BET_ (m^2^ g^–1^)	*V*_total_ (cm^3^ g^–1^)
Fe-MIL-88B	15.3	0.027
Fe_0.7_Ga_0.3_-MOF	26.5	0.058
Fe_0.5_Ga_0.5_-MOF	28.9	0.054
Fe_0.3_Ga_0.7_-MOF	33.4	0.043
Ga-MIL-53	24.2	0.064

a *S*_BET_ = total surface area from BET analysis, *V*_total_ = total pore volume at maximum *p*/*p*_0_.

The thermal behavior under an oxidant atmosphere (Figure 3d and Table S4) helps to examine the stability of the materials under real application circumstances. From the thermograms, it can be seen that the first region, related to the release of water and solvent molecules, represents the lowest weight loss for all materials (<8%). The second region is associated with the loss of structural counterion of the MOF, like –NO_3_^–^ or –Cl^–^. For Fe-MIL-88B, this loss corresponded to 12%, while for Fe_0.3_Ga_0.7_-MOF and the other materials, it was just 8% and 5–3%, respectively. The third region is related to organic linker degradation and, consequently, the collapse of the MOF structure. The Fe-MIL-88B, Fe_0.7_Ga_0.3_-MOF, Fe_0.5_Ga_0.5_-MOF, Fe_0.3_Ga_0.7_-MOF, and Ga-MIL-53 showed a weight loss of 51%, 60%, 62%, 56% and 59%, respectively, throughout this process. Finally, the fourth region exposes the final mass of the decomposed material, which stands out as the metal oxide of the MOF, like Fe_3_O_4_ or Ga_2_O_3_ in the case of Fe-MIL-88B and Ga-MIL-53.^62^ The materials ended up with 29.9–37.5% of the mass at the final temperature of 555 °C, which is consistent with data in the literature.^34,51^ It is worth mentioning that the Ga-MIL-53 also stands out as the most thermal stable material, having the full decomposition of the structure only at 555 °C.

### 3.2 Evaluation of Chemical Stability in Water and Oxidative Environments

The stability of the MOFs was evaluated by exposure to aqueous and oxidative (H_2_O_2_) environments at room temperature (25 °C ± 1). Stability was assessed by measuring the amount of leached metal and analyzing structural data through FT-IR, XRD, and SEM techniques. According to the XRD in Figure 5a, it can be seen that after 7 d in an aqueous environment, the Fe-MIL-88B showed the highest difference in the crystallinity pattern, while other MOFs exposed minor deviations, which may be associated with shifts of the diffraction peaks due to adsorption of water or solvent exchange (host–guest interactions) in the inner structure.^63^ Similar results have been reported previously by Vuong et al. (2013), which showed that the bimetallic FeNi_2_-MIL-88B has its diffraction peaks shifted through immersion in different solvents.^64^ However, for the oxidative environment (Figure 5b), the Fe-MIL-88B structure has highly changed in 1 h, and it was completely collapsed in 24 h. When considering the Fe_*x*_Ga_*y*_-MOFs, it is clear to see that after 1 h (pale colored lines), compared to the pristine material, all MOFs already showed a stable XRD pattern closer to the original one. Besides, after 24 h, all MOFs (except the Ga-MIL-53) showed major changes in their crystallinity, indicating a phase transition. Among the bimetallic, Fe_0.3_Ga_0.7_-MOF had the lower loss in its crystallinity, which is also supported by the fact that Ga-MIL-53 also exhibited outstanding stability even in 24 h of immersion, indicating that the more the Ga^3+^ content, the more stable the structure is. Thus, it is concluded that the water itself does not lead to instability in these frameworks,^65^ and in fact, it is the H_2_O_2_ that reacts with the Fe present in the MOF, which would lead to the collapse of the structure, generating soluble iron (metal) species. Indeed, as the gallium content increases in the material, the peroxidase-like activity decreases due to Ga^3+^ inertness compared to Fe^3+^.^66^ Besides, the micrographs in Figure S12 also corroborate this difference in morphology, caused by loss of crystallinity in oxidative environments and the conservation of structure in an aqueous environment. For example, Fe-MIL-88B (Figure S12a) completely lost its rod-like morphology within 1 h in H_2_O_2_ while Ga-MIL-53 resembles the same (Figure S12e).

**Figure 5:**
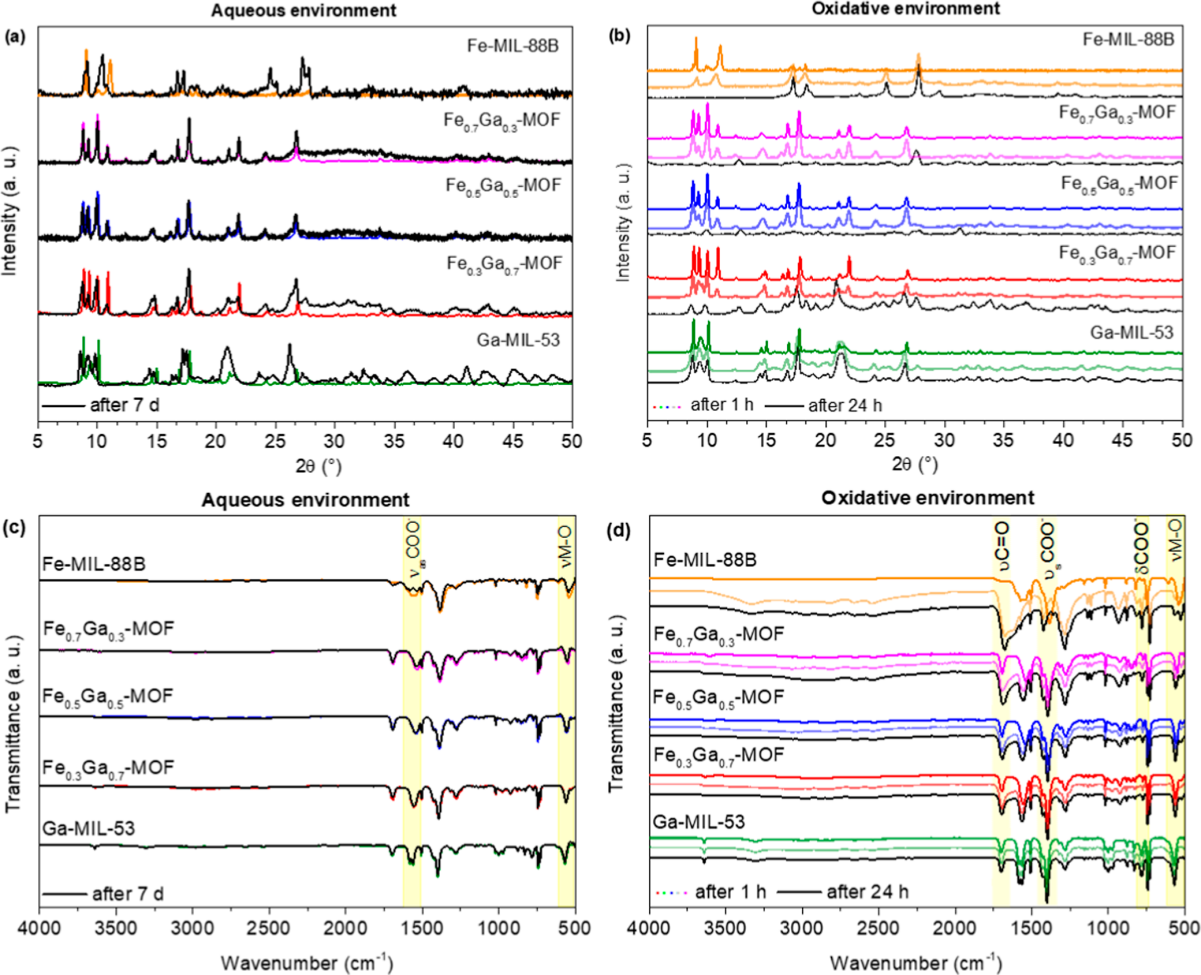
PXRD of the synthesized materials before and after the stability test (a) in an aqueous environment (7 d in immersion) and (b) in an oxidative environment (1 and 24 h in immersion). Comparison of FT-IR spectra of the different materials in (c) aqueous and (d) oxidative environments. Colored straight line: before test/pristine. Faded colored line: after 1 h. Black straight line: after the end of the test.

In terms of the FT-IR spectra, it can be seen that Fe_*x*_Ga_*y*_-MOF and Ga-MIL-53 remained unchanged, whereas Fe-MIL-88B exhibited more pronounced differences in its vibrational bands after aqueous stability tests (Figure 5c). A slight decrease in the bands associated with the carboxylate group and Fe–O bonds was observed for this material. This reduction is most likely the result of interactions with water molecules, which may protonate the –COO^–^ groups and by the low Fe leaching (4.4%), respectively.^67^ However, in an oxidative environment (Figure 5d), every vibration of Fe-MIL-88B is drastically changed, indicating the degradation of the framework structure. This can be highlighted by the M–O band at 547 cm^–1^, which indicates the breaking of Fe–O bonds in the nodes. For Fe_0.3_Ga_0.7_-MOF, a slight intensity loss in the vibrational bands can be seen after 24 h, although the framework spectral pattern is still present. This indicates that just a part of the structure might be degraded, which could be associated with the increasing metal leaching (Fe and Ga). Nevertheless, no significant changes were observed in the Ga-MIL-53 spectra, indicating the higher stability of gallium clusters in the presence of oxidative agents.

The results presented in Figure 6 are the evaluation of metal leaching during chemical stability tests and the iodometric titration used to quantify hydrogen peroxide conversion. In an aqueous environment, Fe-MIL-88B presented a low iron leaching (4.4% Fe), while other materials did not lead to any leaching (0%), thus indicating a more stable framework. In the oxidative environment (Figure 6a), Fe-MIL-88B showed a higher metal leaching (48.0%) in 1 h and was almost fully degraded in 24 h. Fe_*x*_Ga_*y*_-MOFs showed lower metal leaching after 1 h as the gallium content increased. However, in 24 h, these materials also presented high leaching of iron and gallium. This result can be attributed to the presence of mixed metallic nodes, as well as the disruption of Fe–O clusters, which consequently leads to the breakdown of some Ga–O clusters.^68^ Therefore, Ga-MIL-53 showed excellent stability toward all conditions tested with no detected metal leaching, proving the robustness of gallium in the synthesized materials. Figure S13 shows the colors of the liquid phase after material exposure to an oxidative environment, visually indicating the stability condition of these materials.

**Figure 6:**
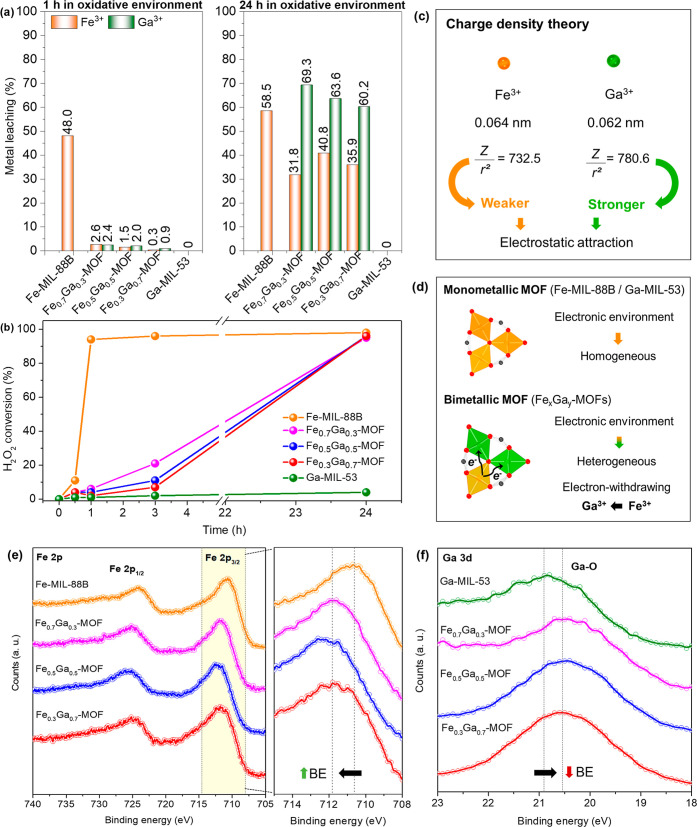
(a) Evaluation of the metal leaching and (b) H_2_O_2_ conversion of the catalysts under an oxidative environment (H_2_O_2_ 0.65 mol L^–1^) at 25 °C. (c) Representation of charge density theory between Fe^3+^ and Ga^3+^. (d) Comparison of mono- and bimetallic MOF nodes in the function of an electronic environment. (e) High-resolution XPS spectra overlay of Fe 2p and (f) Ga 3d in the monometallic MOFs vs bimetallic MOFs. BE = binding energy.

Regarding the decomposition of hydrogen peroxide into Fenton-like products (^•^OH, ^•^OH_2_) and H_2_O + O_2_, it can be seen in Figure 6b that all materials had different activities for this reaction. Fe-MIL-88B, Fe_0.7_Ga_0.3_-MOF, Fe_0.5_Ga_0.5_-MOF, Fe_0.3_Ga_0.7_-MOF and Ga-MIL-53 presented rates of decomposition (*n*H_2_O_2_/time) equals to 6.8, 0.4, 0.3, 0.2, and 0.1 mmol H_2_O_2_ per h, respectively. These decomposition values were equivalent to 94%, 6%, 4%, 2%, and 1% conversion, respectively. However, in 24 h, all materials, except the Ga-MIL-53, presented a conversion of H_2_O_2_ higher than 96%. Furthermore, Fe_0.3_Ga_0.7_-MOF showed to be the most stable of the Fe_*x*_Ga_*y*_-MOF materials, consistently holding both iron and gallium within the structure after 1 h and exhibiting a controlled decomposition of H_2_O_2_, indicating that it would be a viable candidate for catalytic applications, for reactions carried out in not very long periods. All these experiments corroborate the success of structure engineering by switching redox-active Fe^3+^ for redox-inactive Ga^3+^ centers, which used to happen in physiological environment of *P. aeruginosa* and can now be applied to MOFs.^69,70^

In order to deeply understand the enhanced stability of the Fe_*x*_Ga_*y*_-MOFs, it is crucial to consider the higher charge density (*Z*/*r* ^2^) and electron-withdrawing effect within the Fe–O–Ga coordination environment (Figure 6c). Ga^3+^ exhibits a higher charge density than Fe^3+^ (780.6 nm^–1^ vs 732.5 nm^–1^) due to its smaller ionic radius (0.62 Å vs 0.64 Å in octahedral coordination), leading to a stronger electrostatic attraction toward oxygen atoms in the MOF. This results in an electron redistribution effect, where Ga^3+^ withdraws electron density from the Fe^3+^ centers, as shown in Figure 6d.^71^ This electron-withdrawing effect is directly reflected in the XPS analysis (Figure 6e,f). A clear shift in the Fe 2p binding energies toward higher values is observed in the bimetallic MOFs compared to the monometallic Fe-MIL-88B. For example, the Fe 2p_3/2_ peak shifts from 710.8 eV in Fe-MIL-88B to a range of 711.8–712.2 eV in Fe_*x*_Ga_*y*_-MOFs, confirming the electronic effects induced by Ga^3+^ incorporation in the same node. Concurrently, in the Ga 3d spectra, Ga-MIL-53 exhibits the Ga–O bond at 20.9 eV, whereas in Fe_*x*_Ga_*y*_-MOFs, this peak shift slightly lower (20.7–20.6 eV), further supporting the occurrence of Ga^3+^ electron-withdrawing effect.^28,45^ This analysis aligns with previous studies, such as Liu et al. (2016),^46^ which emphasize that changes in the electronic environment observed in high-resolution XPS spectra provide strong evidence of homogeneous mixed metal incorporation within the MOF nodes. Additionally, this electronic redistribution effect may explain the more controlled H_2_O_2_ conversion observed in Fe_*x*_Ga_*y*_-MOFs, as the electron-deficient Fe centers are less susceptible to react.

### 3.3 Catalytic Tests of Methane Oxidation

The impact of the various parameters such as mass of catalyst, temperature, solvent volume, H_2_O_2_ dosage, and the CH_4_ pressure were investigated. The reaction with the observed products is shown in Scheme 1. The Fe_0.3_Ga_0.7_-MOF was used to set these parameters using 1 h of reaction as default and no quantification of CO_2_ was realized at this step in order to optimize the liquid phase products.

**Scheme 1:**
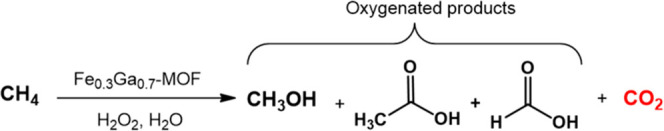
Oxidation of methane catalyzed by Fe_0.3_Ga_0.7_-MOF/H_2_O_2_ and the products observed in this work

Figure 7a shows that among the tested catalyst quantities (20, 35, and 50 mg), the use of 20 mg of MOF yielded the highest productivity (601 μmol g_cat_^–1^), with formic acid, acetic acid, and methanol as the main oxygenated products, respectively, being considered the optimal catalyst mass for this system.

**Figure 7:**
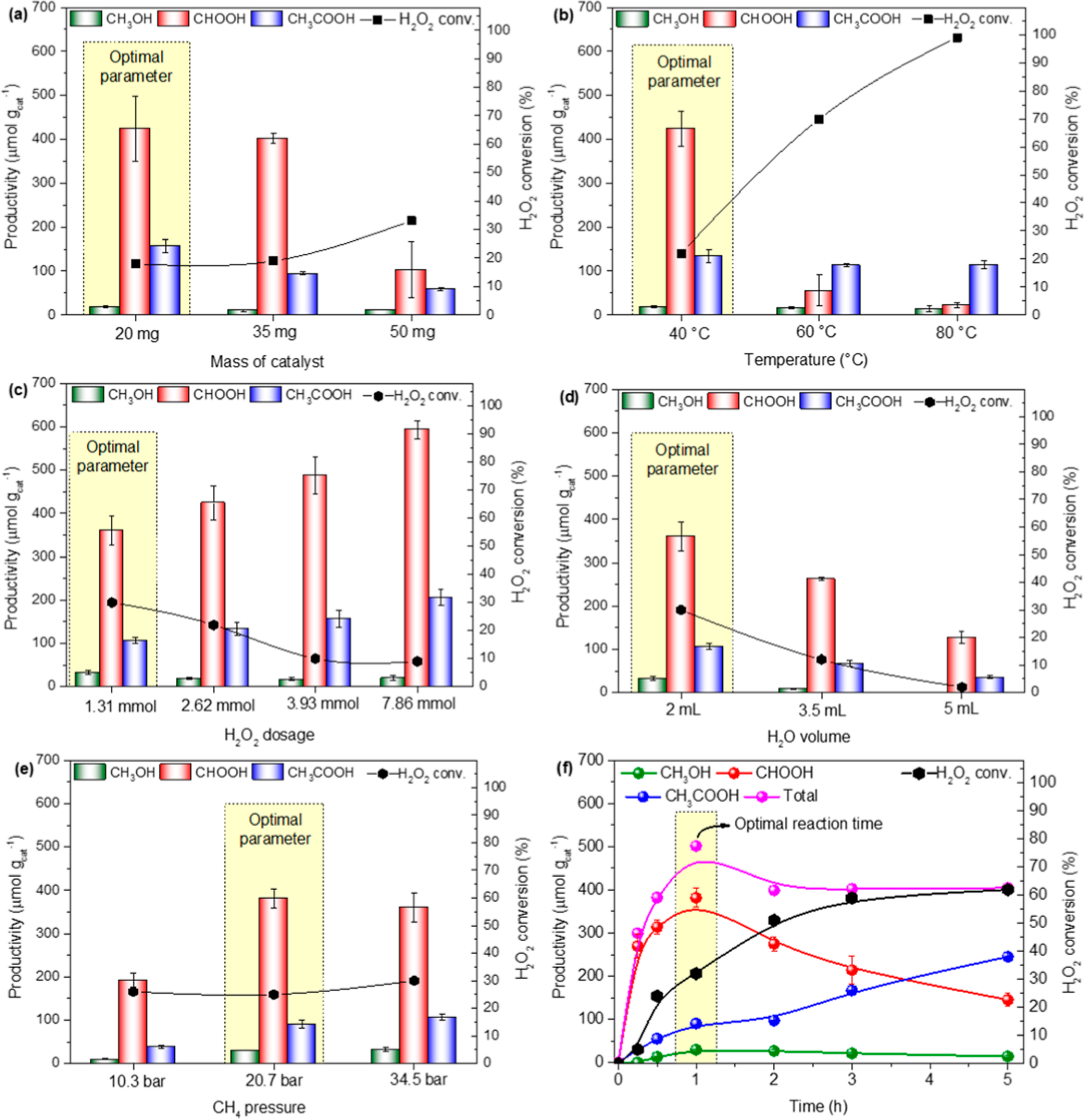
Assessment of catalytic parameters in the direct methane oxidation using Fe_0.3_Ga_0.7_-MOF. (a) Variation of catalyst mass. 2 mL of H_2_O, 2.63 mmol H_2_O_2_, 40 °C, 34.5 bar CH_4_, and 1000 rpm. (b) Variation of temperature. 2 mL of H_2_O, 20 mg of MOF, 2.63 mmol H_2_O_2_, 34.5 bar CH_4_, and 1000 rpm. (c) Variation of oxidant dosage. 2 mL of H_2_O, 20 mg of MOF, 40 °C, 34.5 bar CH_4,_ and 1000 rpm. (d) Variation of water volume. 20 mg of MOF, 1.31 mmol H_2_O_2_, 40 °C, 34.5 bar CH_4,_ and 1000 rpm. (e) Variation of methane pressure. 2 mL of H_2_O, 20 mg of MOF, 1.31 mmol H_2_O_2_, 40 °C, and 1000 rpm. (f) Evaluation of different reaction times using 2 mL of H_2_O, 20 mg of MOF, 1.31 mmol H_2_O_2_, 40 °C, 20.7 bar CH_4_, and 1000 rpm.

In terms of temperature (Figure 7b), the highest productivity was achieved at 40 °C. At this temperature, H_2_O_2_ consumption was lower (22%), indicating a more controlled conversion of the oxidizing agent. On the other hand, when the temperature was increased to 80 °C, the productivity decreased to 153.2 μmol g_cat_^–1^. This observation may be attributed to the nearly complete consumption of H_2_O_2_ (99% conversion), leaving less oxidant available for methane oxidation or because of overoxidation of the products at higher temperatures.

As depicted in Figure 7c, testing different amounts of H_2_O_2_ revealed that a lower concentration (1.31 mmol) led to a lower overall productivity (500.7 μmol g_cat_^–1^). In contrast, increasing the H_2_O_2_ dosage (2.62, 3.93, and 7.86 mmol) led to higher total productivities (577.6, 662.0, and 820.8 μmol g_cat_^–1^). This trend, commonly reported in similar studies, arises because methane oxidation relies on C–H activation, and the higher oxidant concentrations promotes this process.^72^ Although a higher H_2_O_2_ dosage resulted in better productivity, catalyst stability is also a key factor. After removing the catalyst from the reaction, the test with lower H_2_O_2_ remained a colorless solution, indicating no or negligible Fe leaching, while higher concentrations led to a pale-yellow solution, signaling Fe leaching from the MOF. These results highlight the parameter dependence effects on both H_2_O_2_ conversion, productivity, and the stability of the catalyst system.

The fourth variable studied was the solvent volume, as shown in Figure 7d. It was observed that using 2 mL of H_2_O as the solvent resulted in the highest productivity (500.7 μmol g_cat_^–1^) compared to using 3.5 and 5 mL of H_2_O. This outcome can be attributed to the higher concentration of reactants and the increased likelihood of the MOF’s active sites reacting at the liquid–gas–solid interface, considering methane’s low solubility in water.^73^

In Figure 7e it can be seen that CH_4_ pressure had a significant impact on reaction productivity. At 34.5 and 20.7 bar, the total productivities were nearly identical (500.8 and 501.7 μmol g_cat_^–1^, respectively), indicating that beyond a certain pressure, increasing methane availability does not substantially enhance the reaction. However, at 10.3 bar, productivity dropped sharply to 241.7 μmol g_cat_^–1^, likely due to the lower dissolved methane in water at reduced pressures.^74^ Notably, H_2_O_2_ consumption was unaffected by the CH_4_ pressure.

Based on the optimized reaction conditions, experiments were conducted at varying reaction times to determine the maximum productivity (Figure 7f). Methanol (MeOH) and formic acid (FA) achieved their highest productivity at 1 h of reaction. After this point, acetic acid (AA) production began to increase, while both MeOH and FA exhibited a gradual decline in productivity. This decline may be attributed to the continuous overoxidation of products, like methanol into formic acid, acetic acid and/or carbon dioxide.^75^ After this time, productivity slightly decreases and stabilizes, indicating that the optimized reaction time would be 1 h, presenting a productivity of 29.9, 381.9, and 90.1 μmol g_cat_^–1^ for MeOH, FA, and AA respectively.

Additionally, some control experiments were realized, as seen in Table S5. When the reaction was carried out without the MOF (entry 6), only 1% H_2_O_2_ conversion was observed after 1 h of reaction with a maximum conversion of 8% after 5 h (entry 7). In contrast, when the Fe_0.3_Ga_0.7_-MOF was present, a remarkable 62% conversion of H_2_O_2_ was achieved within 5 h (entry 3), with 31% conversion achieved after just 1 h (entry 2). No additional MeOH, FA, or AA was produced in the absence of the catalyst (entries 6 and 7), compared to the reaction using MOFs (entries 1–3). Also, the same behavior was seen when the reaction was made by substituting CH_4_ with argonium (Ar), indicating that the products came from the CH_4_. Due to the inert atmosphere, the H_2_O_2_ conversion drastically reduced from 31% to 7% (entries 2 and 5). The MOF, besides having C in its structure, does not act as a source of it, and the increase in the liquid phase productivity relies on the methane.

After finding the optimized reaction parameters, the performance of the pristine and Fe_0.3_Ga_0.7_-MOF was compared considering the products in the liquid and gas phase. Figure 8a shows that Fe-MIL-88B displayed notable productivity of methanol (53.5 μmol g_cat_^–1^), formic acid (578.2 μmol g_cat_^–1^), acetic acid (245.2 μmol g_cat_^–1^), and alongside an undesired high quantity of CO_2_ (11,335.4 μmol g_cat_^–1^). However, it also exhibited the highest level of instability under the reaction conditions, leading to its dissolution and leaching into the reaction media. This suggests that the amount of CO_2_ produced could be also from the organic linker oxidation.^76^ Also, it is noted that the bimetallic structure reduced the CO_2_ evolution by approximately 10,700 μmol g_cat_^–1^ (95%), compared to the pristine MOF. This fact could be related to more isolated, stable, and lesser active sites compared to the high iron content in Fe-MIL-88B.^12^ In regard to the distribution of oxygenated products (Figure 8b), the bimetallic Fe_0.3_Ga_0.7_-MOF exhibited a higher value for formic acid (76% vs 66%), a lower one for acetic acid (18% vs 28%), and the same for methanol (6%) when compared with the Fe-MIL-88B. Meanwhile, Ga-MIL-53 did not show any formation of products and a minimal H_2_O_2_ conversion (4%). Remarkably, it can be seen in Figure 8c that the Fe_0.3_Ga_0.7_-MOF is stable throughout three cycles without any significant loss in activity or product distribution (<10%), indicating the successful stabilization of the catalyst through gallium doping.

**Figure 8:**
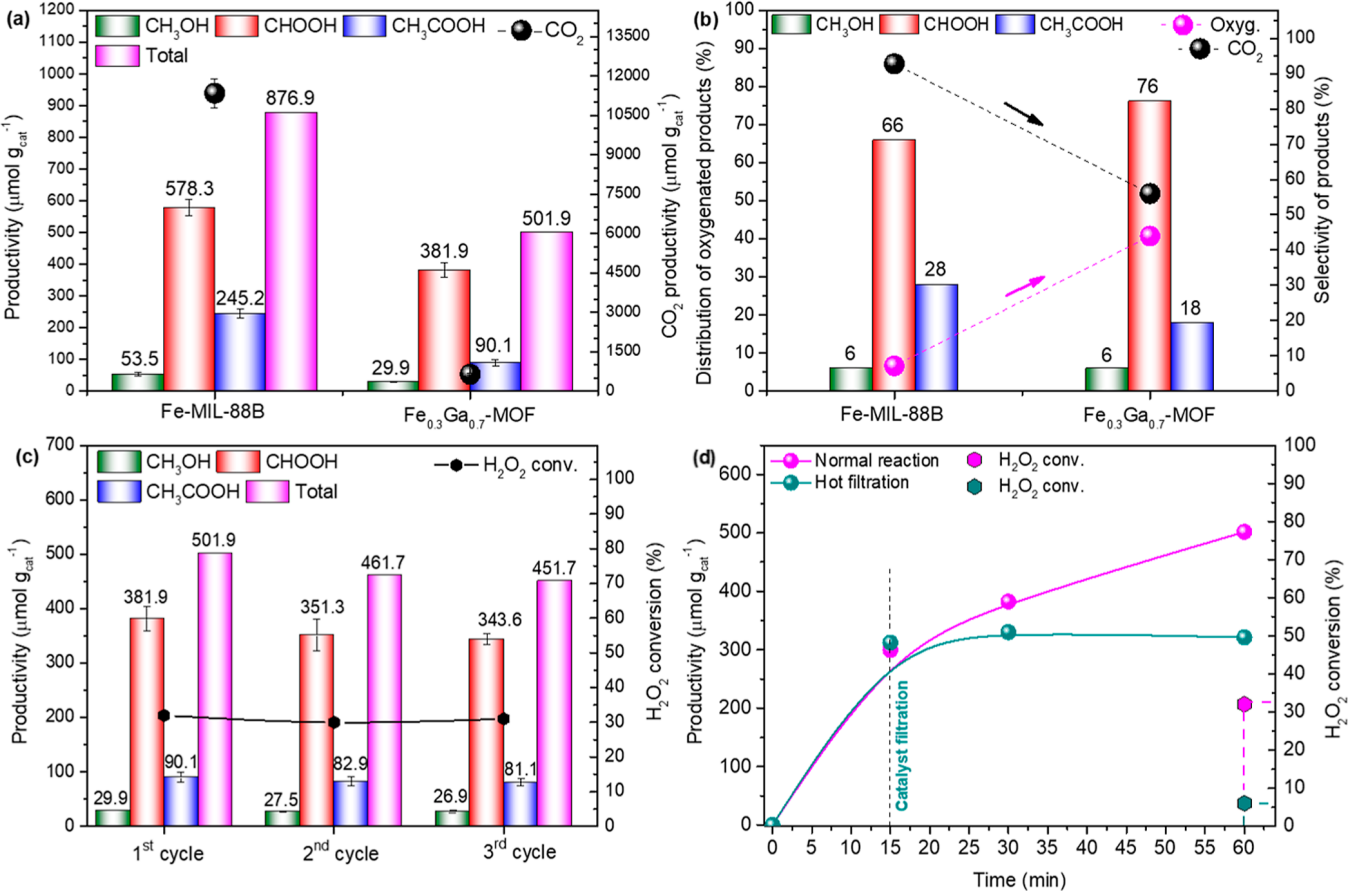
(a) Comparison of productivity (oxygenates and CO_2_) between the Fe-MIL-88B and Fe_0.3_Ga_0.7_-MOF. (b) Comparison of the selectivity parameter and distribution of products between the Fe-MIL-88B and Fe_0.3_Ga_0.7_-MOF. (c) Recyclability test for methane oxidation using Fe_0.3_Ga_0.7_-MOF in 1 h of reaction. (d) Hot filtration test for methane oxidation using Fe_0.3_Ga_0.7_-MOF.

The heterogeneity of the active species was studied using a hot filtration experiment (Sheldon’s test^31^) (Figure 8d). The results show that product formation ceased when the catalyst was filtered out of the reaction after 15 min. Also, no metal leaching was detected for Fe_0.3_Ga_0.7_-MOF, concluding that the conversion of methane occurs heterogeneously through the metal species present within the MOF structure.

Regarding the postreaction characterization of the materials, Figure S14 shows the metal content after the catalytic tests, allowing the confirmation of iron leaching from Fe-MIL-88B (38.3%) and the stability of the Fe_0.3_Ga_0.7_-MOF (0.1% Fe leaching in third cycle) and Ga-MIL-53 (0%). Analyzing the XRD (Figure S15a), it is noted that the Fe-MIL-88B loses most of its crystallinity, while Fe_0.3_Ga_0.7_-MOF goes through a phase transition, retaining the major diffraction peaks from 5 to 20°. Ga-MIL-53 diffraction patterns remain unchanged, presenting some minor differences, probably due to its flexible characteristic and host–guest interactions. FT-IR spectra (Figure S15b) expose some changes in the Fe-MIL-88B pattern related to the bands of C=O and COO^–^ stretching (1684 and 1284 cm^–1^, respectively), which suggests organic linker modification in the structure. Besides, Fe_0.3_Ga_0.7_-MOF and Ga-MIL-53 remain with the same profile characteristics. SEM analysis (Figure S16) exposes the Fe-MIL-88B deformation into small aggregates postreaction, whereas Fe_0.3_Ga_0.7_-MOF and Ga-MIL-53 retain their original particle morphology.

Furthermore, when comparing different methane oxidation studies in the literature (Table S6), our work in developing the Fe_0.3_Ga_0.7_-MOF stands out with a notable productivity that surpasses many of the previously reported catalysts and achieves a FA oxygenate distribution of 79%. Moreover, the mild reaction conditions used in this study, 40 °C, 1 h reaction time, and 20.7 bar of CH_4_, represent an improvement in terms of energy efficiency, compared to the harsher conditions typically employed. While previous studies have predominantly focused on catalytic efficiency and product selectivity, the stability of catalysts under reaction conditions remains an often overlooked aspect. We addressed this critical gap by evaluating the catalyst performance up to three consecutive cycles. However, for the practical use of these materials and their transition from laboratory-scale research to industrial applications, long-term stability assessments are essential. Furthermore, a deeper understanding of the structural changes occurring in the materials during the reaction is necessary to guide future developments.^1^

### 3.4 Mechanistic Insights for the Methane Oxidation

To investigate the mechanism of the methane oxidation, some tests were performed to better understand the formation of the products. The tests involve the addition of quenching agents in the methane oxidation to identify what reactive oxygen species (ROS) are involved in the process. Thus, salicylic acid (SA) and the *p*-benzoquinone (pBQ) were used as quenchers of ^•^OH and ^•^OOH/^•^O_2_^–^ respectively.^77^ Figure 9a shows that the formation of the oxygenated products is highly dependent on the formation of these radicals (^•^OH, and ^•^OOH/^•^O_2_^–^), leading to a reduction of nearly to 92% in the productivity when the scavengers are present for Fe-MIL-88B and Fe_0.3_Ga_0.7_-MOF. This result shows the decisive role of radicals to the oxygenate production and also the similarity of the catalytic mechanism. Along with the generation of ROS (eqs 1 and 2), there is also another route of H_2_O_2_ decomposition, which is called catalase (eq 3), which can compete with this.

1 H2O2→2OH·

2 H2O2+OH·→OOH·+H2O

3 $$H_{2}O_{2}\to 1/2O_{2}+2H_{2}O$$

**Figure 9:**
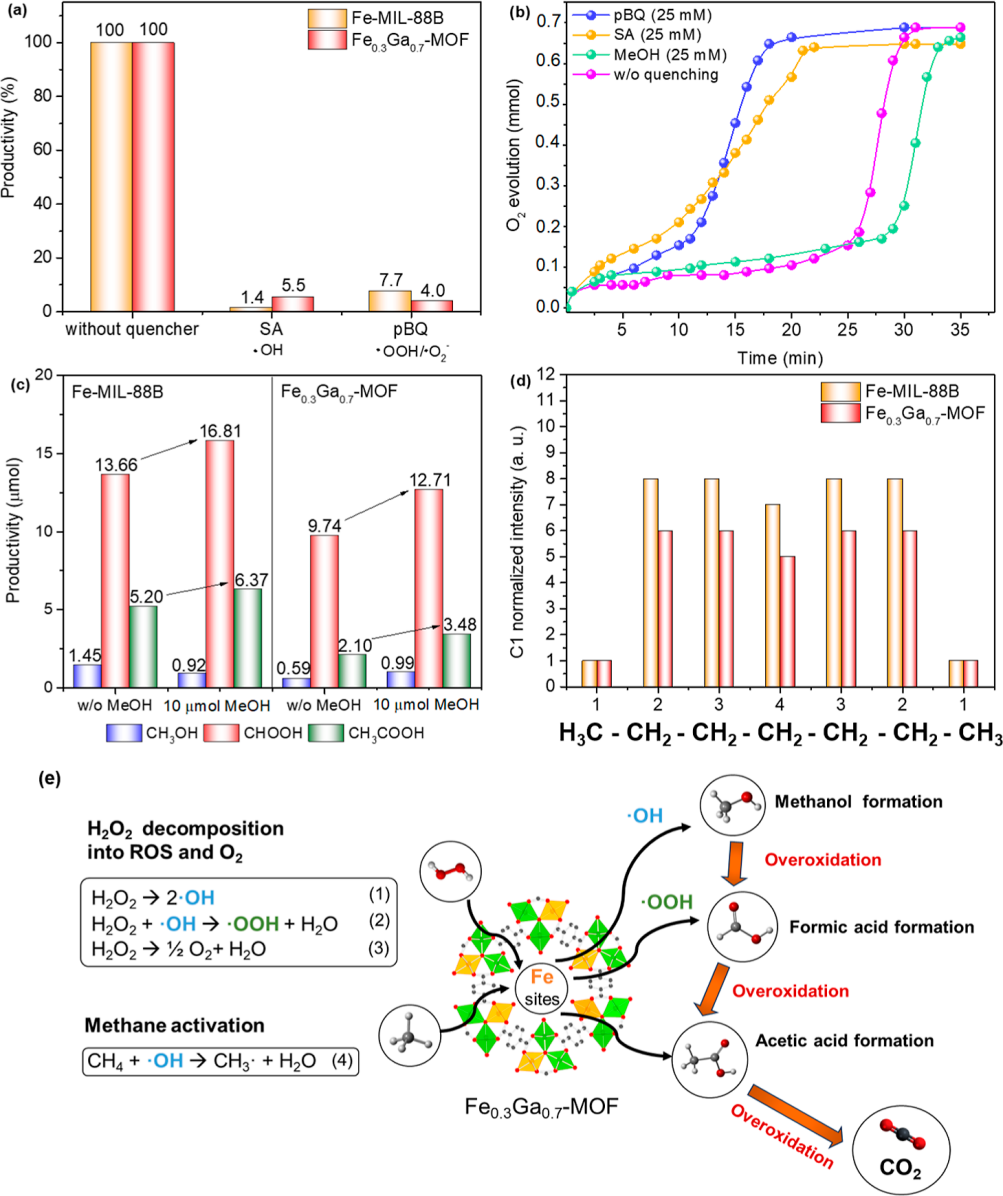
(a) Productivity in methane oxidation using ROS quenching agents. Reaction conditions: 1 h, 2 mL of H_2_O, 20 mg of MOF, 1.31 mmol H_2_O_2_, 40 °C, [quencher] = 25 mM, 20.7 bar CH_4_, and 1000 rpm. It was considered that the reaction without quenchers has a productivity equivalent to 100% for easier comparison. (b) O_2_ production in H_2_O_2_ decomposition in an adiabatic system using the Fe-MIL-88B. Reaction conditions: 2 mL of H_2_O, 20 mg of MOF, 1.31 mmol H_2_O_2_, 40 °C, SA, pBQ or MeOH = 25 mM, and 1000 rpm. (c) Test of addition of methanol in the methane oxidation system. Reaction conditions: 1 h, 2 mL of H_2_O, 20 mg of MOF, 1.31 mmol H_2_O_2_, 40 °C, MeOH = 10 μmol in the beginning of the reaction, 20.7 bar CH_4_, and 1000 rpm. (d) Regioselectivity in *n*-heptane oxidation in similar conditions of the methane oxidation. Reaction conditions: 1 h, 2 mL of acetonitrile, 20 mg of MOF, 1.31 mmol H_2_O_2_, 40 °C, 2.75 mmol *n*-heptane, and 1000 rpm. (e) Schematic representation of possible paths on methane oxidation using H_2_O_2_ as oxidant toward the formation of oxygenated products and CO_2_.

To evaluate the competition between the reactions, the O_2_ production was evaluated in the adiabatic system (Figure S3) and the Fe-MIL-88B was used as a model catalyst in the absence of methane (Figure 9b). First, the raw reaction of H_2_O_2_ decomposition in water shows that it takes almost 30 min until the full evolution of O_2_ is over. Meanwhile, when low concentrations of both quenching agents (SA and pBQ) are introduced in the system (black and red lines), the evolution of the O_2_ is accelerated by 10 min. This acceleration suggests that the sequestration of radicals makes the Fenton-like pathway less favorable, favoring a catalase-like reaction instead. However, when methanol is added at the start of the process, the evolution of the O_2_ species slows slightly (by about 5 min), likely due to radical generation and potential overoxidation of methanol. This observation indicates a competitive interaction between the reactions in the presence of substrate.^78^ In this sense, to also explore methanol overoxidation, an additional catalytic test was made through the addition of methanol in the methane oxidation system (Figure 9c). It can be noted that for both MOFs, the quantity of FA and AA was increased in the test compared to the reaction without methanol. This means that the acetic acid and formic acid obtained in the reaction can be formed from the deep oxidation of methanol.^79^

To complement the previous findings, we selected a longer-chain alkane, *n*-heptane, which is more readily oxidizable to strengthen our results. The oxidation of *n*-heptane to isomeric heptanol alcohols was carried out under reaction conditions similar to those used for methane oxidation (Figure 9d). By the analysis of the product distribution, it is revealed that the catalytic system may involve the formation of hydroxyl radicals (^•^OH), which are highly reactive and do not discriminate between hydrogens attached to different carbons in a molecule.^80^ In the case of *n*-heptane, which has four distinct types of carbons numbered 1 to 4, each with a specific number of hydrogens, these radicals do not differentiate between hydrogens attached to carbons C1, C2, C3, and C4. Considering that oxidation processes involve the substitution of a hydrogen atom with oxygen, it is necessary to normalize reactivity based on the number of hydrogens attached to each carbon before comparing the reactivities of different carbons. Then, the regioselectivity parameters were calculated relative to the total alcohols of *n*-heptane formed. The values obtained for Fe-MIL-88B and Fe_0.3_Ga_0.7_-MOF were C1/C2/C3/C4 = 1:8:8:7 and 1:6:6:5, indicating the similarity between the mechanisms of the synthesized catalysts. A standard value for comparison is the regioselectivity obtained in *n*-heptane oxidation with H_2_O_2_–UV, which is known to generate hydroxyl radicals. In this case, the regioselectivity is C1/C2/C3/C4 = 1:7:6:7.^81^ On the other hand, Mn-TMTACN-based systems, also studied by our research group, oxidize via oxo and peroxo groups without generating hydroxyl radicals. These systems exhibit very high regioselectivity, with values of C1/C2/C3/C4 = 1:46:46:35.^82^

Overall, the previous results corroborate the existing literature, supporting the proposed reaction pathway illustrated in Figure 9e. In methane oxidation using an Fe-based MOF catalyst, H_2_O_2_ is first decomposed by the Fe active sites into active oxygen species, such as ^•^OH and OOH^•^. These species then activate methane (CH_4_) by abstracting a hydrogen atom, forming a methyl radical (^•^CH_3_). The methyl radical reacts with ^•^OH to produce methanol (CH_3_OH). Methanol can be further oxidized to formic acid (HCOOH) and acetic acid (CH_3_COOH) through intermediate steps involving additional active oxygen species. Overoxidation of methanol and formic acid leads to the formation of acetic acid and carbon dioxide (CO_2_).^10,83^ Moreover, future research will focus on the spectroscopy, computational details, and *in situ* characterization of these species to gain deeper mechanistic insights. These studies, particularly using Fe–Ga-MOFs, are crucial for advancing our understanding of the reaction dynamics and enhancing the catalyst design.

## 4 Conclusions

This study demonstrated that through structural engineering, incorporating Ga^3+^ doping into the synthesis of Fe-MIL-88B significantly enhanced the catalyst’s stability in oxidative environments. It was confirmed that the gallium species effectively hindered the decomposition of H_2_O_2_, mimicking some gallium-based complexes in the physiological environment of *P. aeruginosa*, thereby tailoring the iron­(III) peroxidase-like cycle. The chemical stability studies proved to be crucial in evaluating the preservation of the MOF’s crystalline structure following the use of hydrogen peroxide, a commonly employed oxidizing agent. The catalytic investigations revealed that Fe_0.3_Ga_0.7_-MOF is well-suited for the direct oxidation of methane under mild reaction conditions by using H_2_O_2_ in the aqueous phase. Productivities of 29.9, 381.9, and 90.1 μmol g_cat_^–1^ were obtained for methanol, formic acid, and acetic acid, which were comparable and even showed better performance compared to those of the existing literature. The total heterogeneity of the catalysis and recyclability for up to three cycles without any significant loss in activity was achieved. The methane oxidation mechanism tests proved the involvement of the decomposition of H_2_O_2_ into reactive oxygen species that activate the MOF and the overoxidation of products. Our work also emphasizes the importance of ensuring and studying catalyst stabilization, conducting postcharacterization after the catalysis which are often overlooked in MOFs studies. Therefore, unlike many conventional approaches that rely on multistep synthesis or postsynthetic modifications, our one-pot synthesis method offers a simpler, more sustainable route to enhance the MOF’s stability.

## Supplementary Material



## Data Availability

Data are contained within the article.

## Notes

Further sample characterizations (FT-IR, SEM, RAMAN, XPS, XRD), scheme of methodology, additional control experiments, and literature comparison over different materials for methane oxidation (PDF)
