# Supplementary material for: Bimetallic (Fe–Ga) Metal–Organic Frameworks for Tailoring Peroxidase-Like Activity: An Approach for Methane Partial Oxidation
Source: ACS Mater Au. 2025 Jul 2;5(5):831–48. doi: 10.1021/acsmaterialsau.5c00045 (PMC12426786; doi:10.1021/acsmaterialsau.5c00045)
Supplement: Supplementary file 1 [file mg5c00045_si_001.pdf]

Supporting Information

Bimetallic (Fe-Ga) Metal-Organic Frameworks for Tailoring Peroxidase-Like Activity: An Approach for Methane Partial Oxidation

Gustavo Felix Bitencourt[a,b], Luana dos Santos Andrade[a], Wandson Lukas do Nascimento Amorim[a], Herich Henrique Lafayete Bastos Lima[a], Gabriela Tuono Martins Xavier[a], José Javier Sáez Acuña[a], Wagner Alves Carvalho[a], Mohamad El Roz[b], Thiago de Melo Lima[c], Dalmo Mandelli[a*]

[a] Universidade Federal do ABC – UFABC, Centro de Ciências Naturais e Humanas, Avenida dos Estados, 5001, Santo André / SP, 09210-580, Brazil

[b] University of Caen, UNICAEN, ENSICAEN, CNRS, Laboratoire Catalyse et Spectrochimie, Bd Maréchal Juin, 6, Caen / Calvados, 14200, France

[c] Universidade Federal Fluminense - UFF, Instituto de Química, Outeiro de São João Batista, s/n, Niterói / RJ, 24020-140, Brazil

**Correspondence author: dalmo.mandelli@ufabc.edu.br*

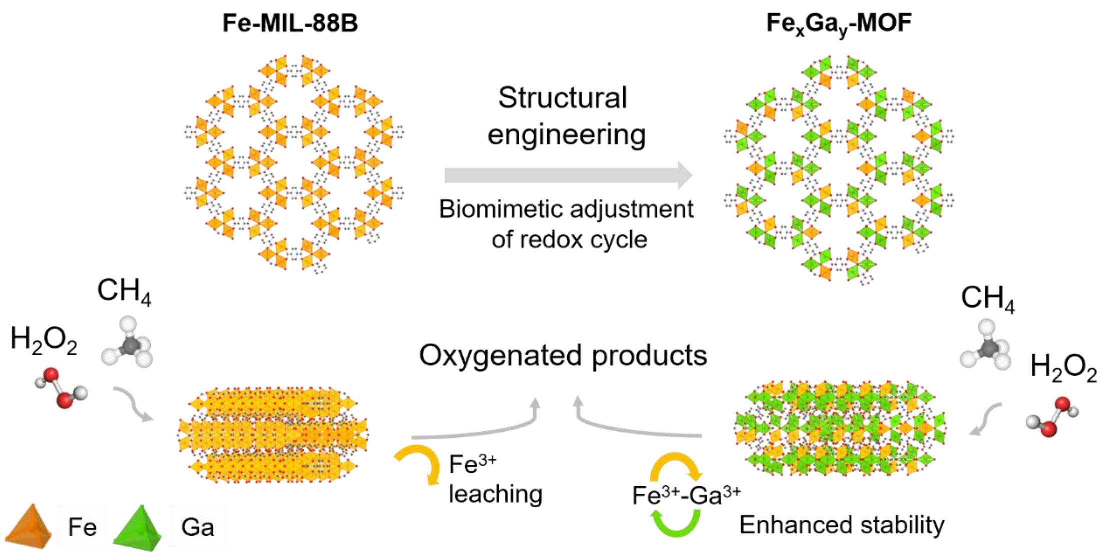

Figure S1. Scheme of structural engineering for the preparation of novel bimetallic metal-organic frameworks for methane partial oxidation.

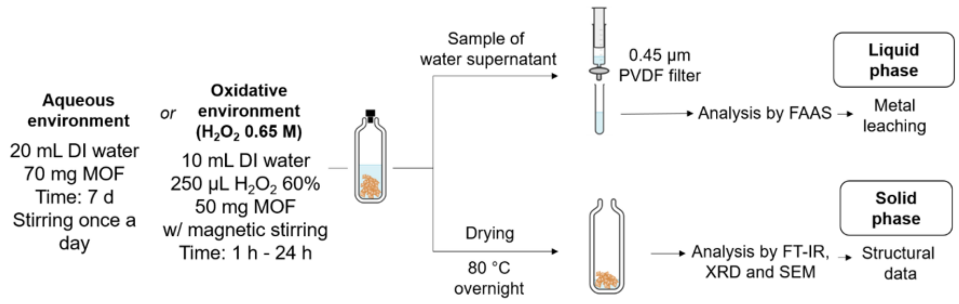

Scheme S1. Methodology of chemical stability tests in aqueous and oxidative environment and sampling process.

For the quantification of CO_2 in gas phase the following procedure was utilized. After the end of the reaction, a handmade junction with a T-valve and a rubber septum was inserted in the end of the outlet valve (J) and the gas phase was relieved through the output valve, maintaining a constant flux viewed by the gas evolution in the water bottle (L). After this, the aliquots were taken with a gas high-pressure syringe and inserted in the GC-MS in the SIM Mode.

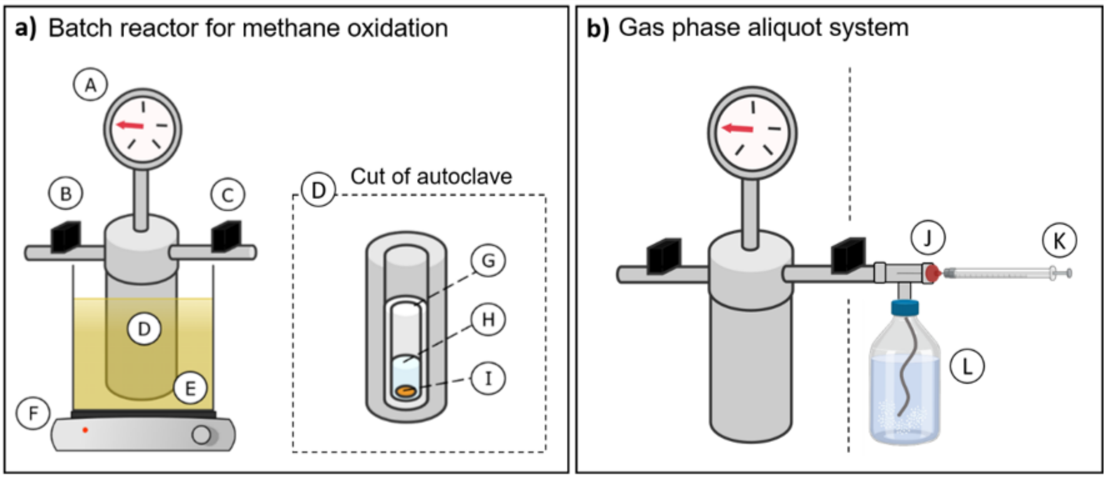

Figure S2. a) Batch reaction system used in the oxidation of methane and **b)** system for collecting gas sample from reaction. A – Manometer; B – CH_4 inlet valve; C – CH_4 output valve; D – Autoclave containing Teflon reactor; E – Oil bath; F – Heating plate with magnetic stirring; G – Teflon reactor; H – Reaction media; I – Catalyst; J – Connection put in the end of the output valve with a septum for taking aliquots; K – High-pressure gas syringe; L – Bottle containing water

The O_2 evolution was made in this apparatus consisted of a 50 mL graduated burette connected at the top to a separatory funnel. Both the burette and the funnel were filled with water, and the displacement of the water column in the burette during the reaction was measured. As the water column was displaced, the position of the separatory funnel was adjusted to maintain the internal pressure equal to atmospheric pressure. The reaction was monitored until no further displacement of the water column in the burette was observed, indicating its end [1].

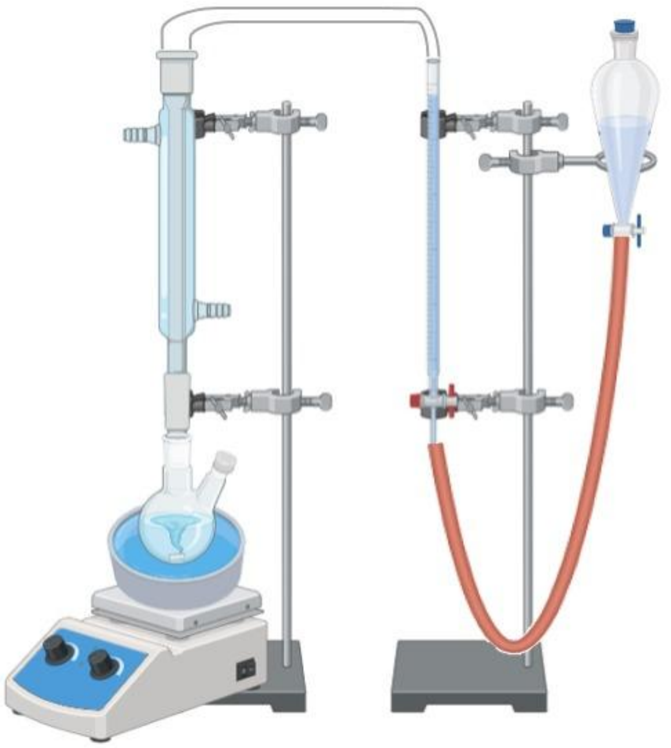

Figure S3. Adiabatic system for measurement of O_2 evolution through the H_2O_2 decomposition. The O_2 evolved can be measured by the burette at the right part of the image. Reaction conditions: 2 mL H_2O, 20 mg MOF, 1.31 mmol H_2O_2, 40 ºC, Salicylic acid, p-benzoquinone or methanol = 25 mM, and 1000 rpm.

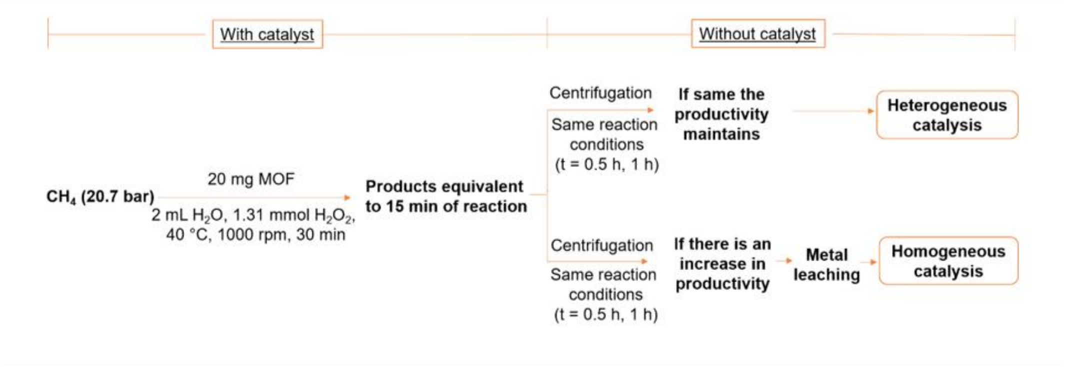

Scheme S2. Scheme of hot filtration test methodology for catalytic tests.

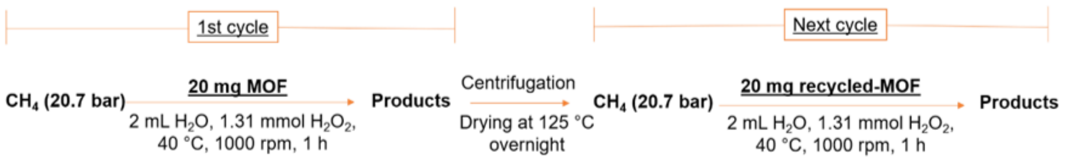

Scheme S3. Scheme of recycling methodology for catalytic tests.

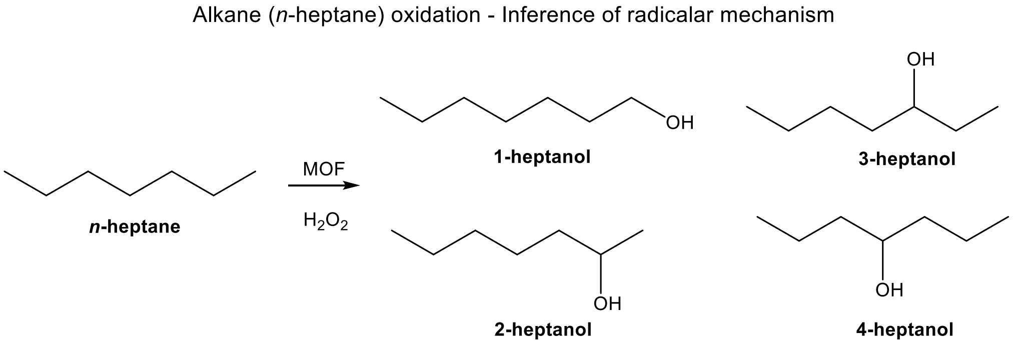

Scheme S4. Reaction of n-heptane oxidation with the synthesized MOFs towards isomeric alcohols. Reaction conditions: 2 mL acetonitrile, 20 mg MOF, 1.31 mmol H_2O_2, 40 ºC, 2.75 mmol *n*-heptane, and 1000 rpm.

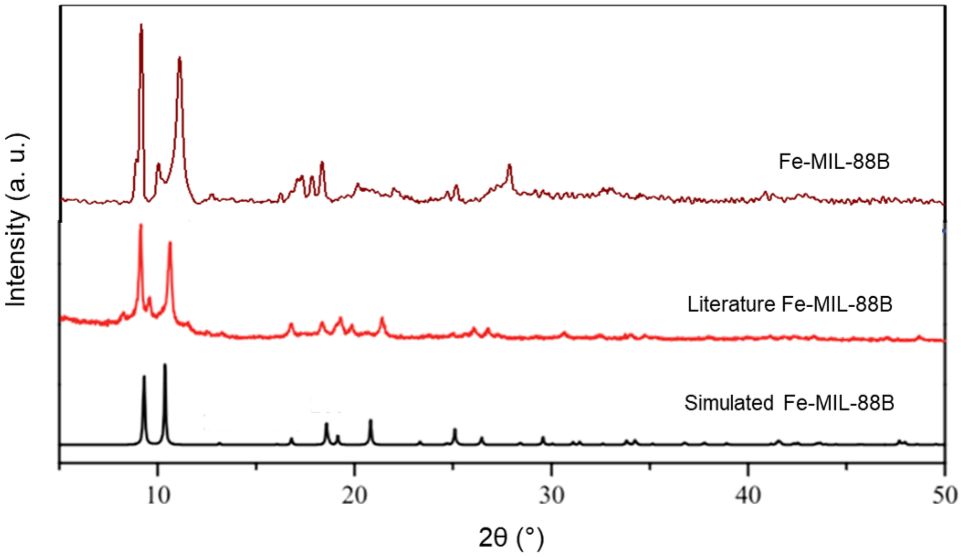

Figure S4. XRD of Fe-MIL-88B synthesized in this work compared to the Fe-MIL-88B synthesized (red line) and simulated (CCDC Code: 285810) (black line) by Liu, J. et. al (2021) [2].

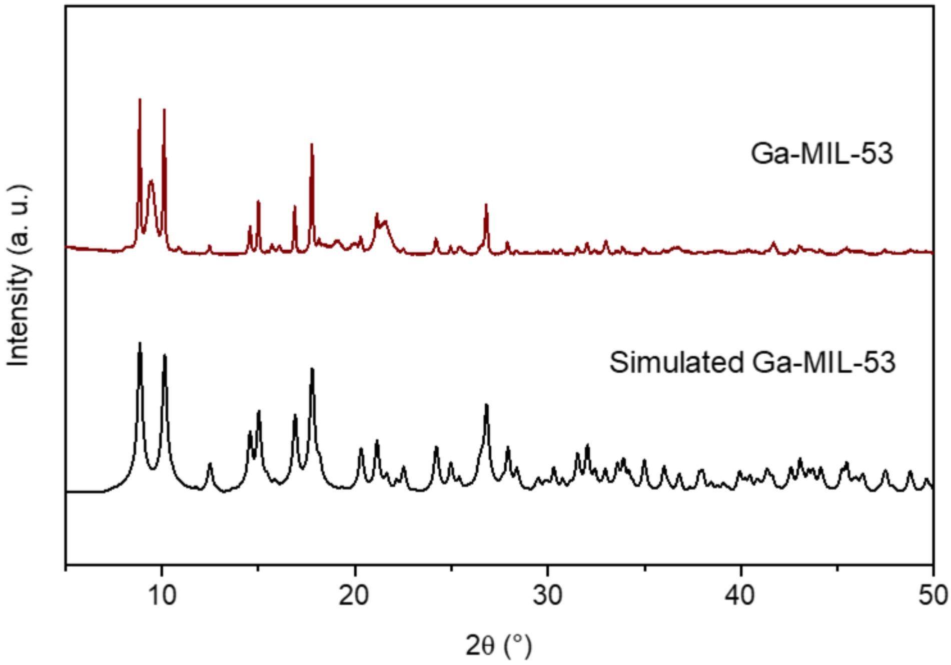

Figure S5. XRD of Ga-MIL-53 synthesized in this work (brown line) compared to the Ga-MIL-53 (CCDC Code: 704888) simulated by Volkringer, C. et. al (2009) [3].

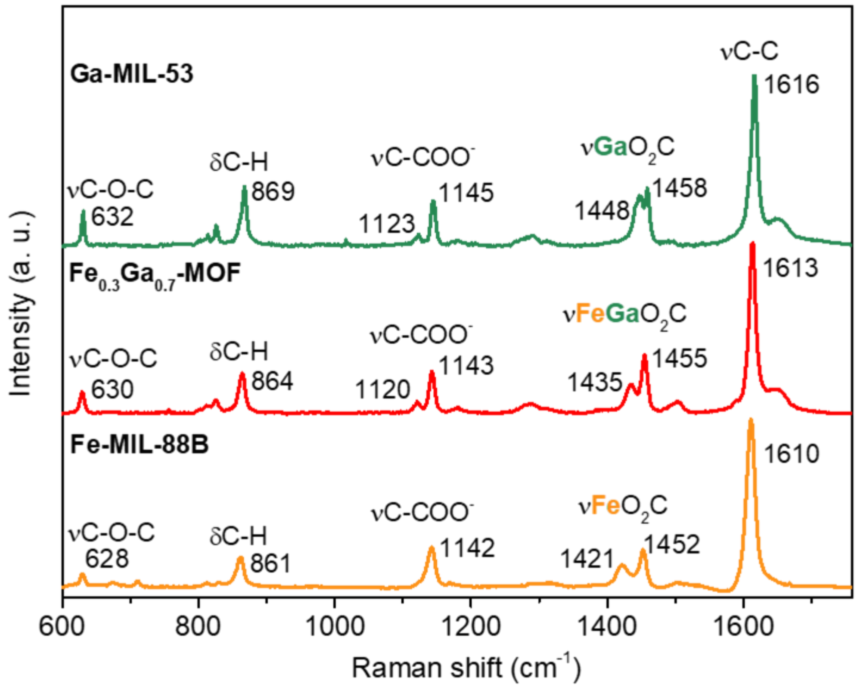

Figure S6. Raman spectra of the monometallic MOFs (Fe-MIL-88B and Ga-MIL-53) in comparison with the bimetallic $Fe_{0.3}Ga_{0.7}$-MOF.

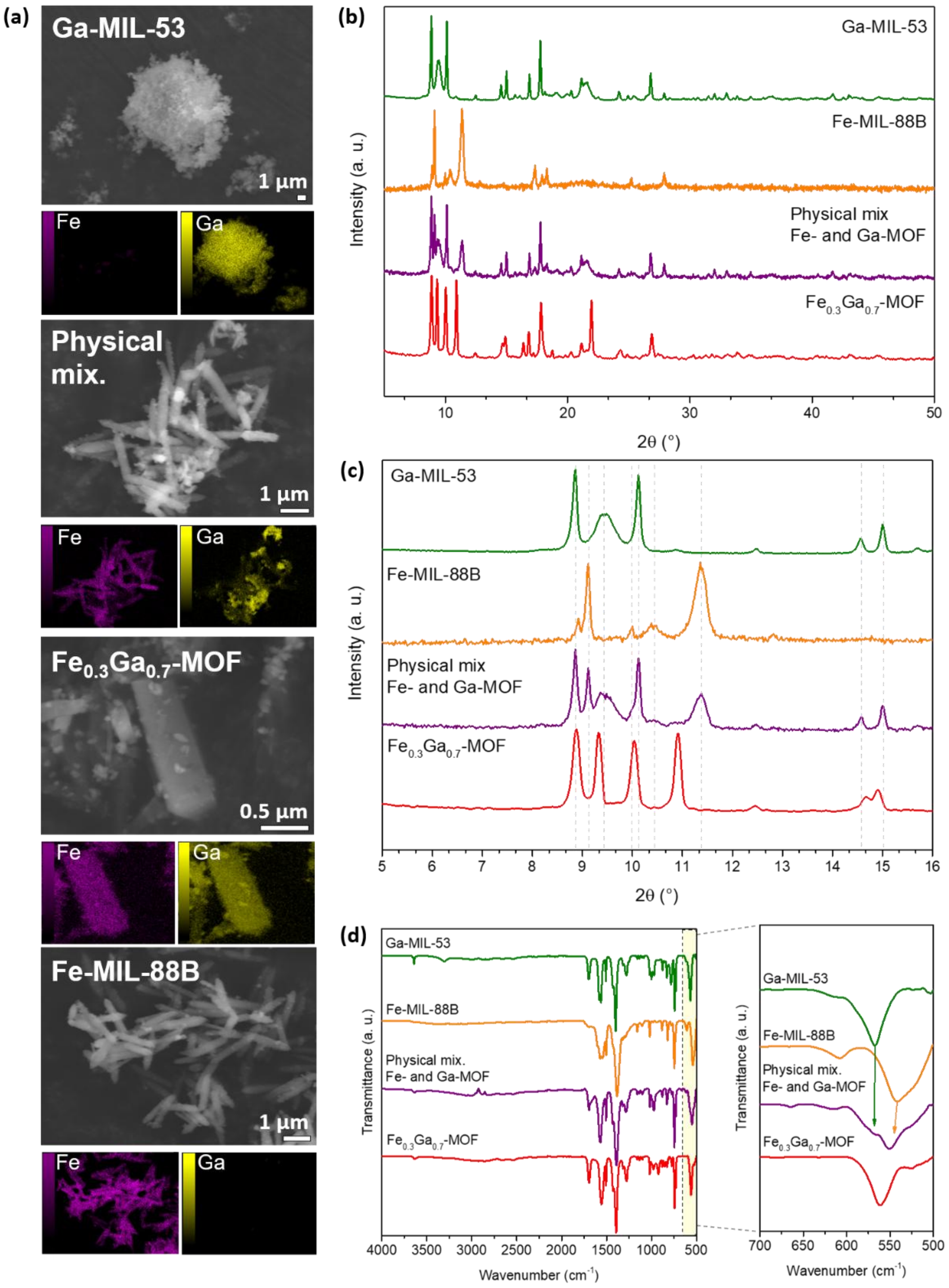

Figure S7. Comparison of pristine MOFs, bimetallic MOF, and physical mixture of Fe-MIL-88B:Ga-MIL-53. **(a)** Mapping of Fe and Ga obtained by SEM-EDS **(b)** XRD from 5º to 50º and **(c)** Main XRD region of materials highlighting similarity between pristine MOFs vs. physical mixture and the difference between $Fe_{0.3}Ga_{0.7}$-MOF **(d)** FT-IR spectra of the materials and amplification of the M-O stretching region.

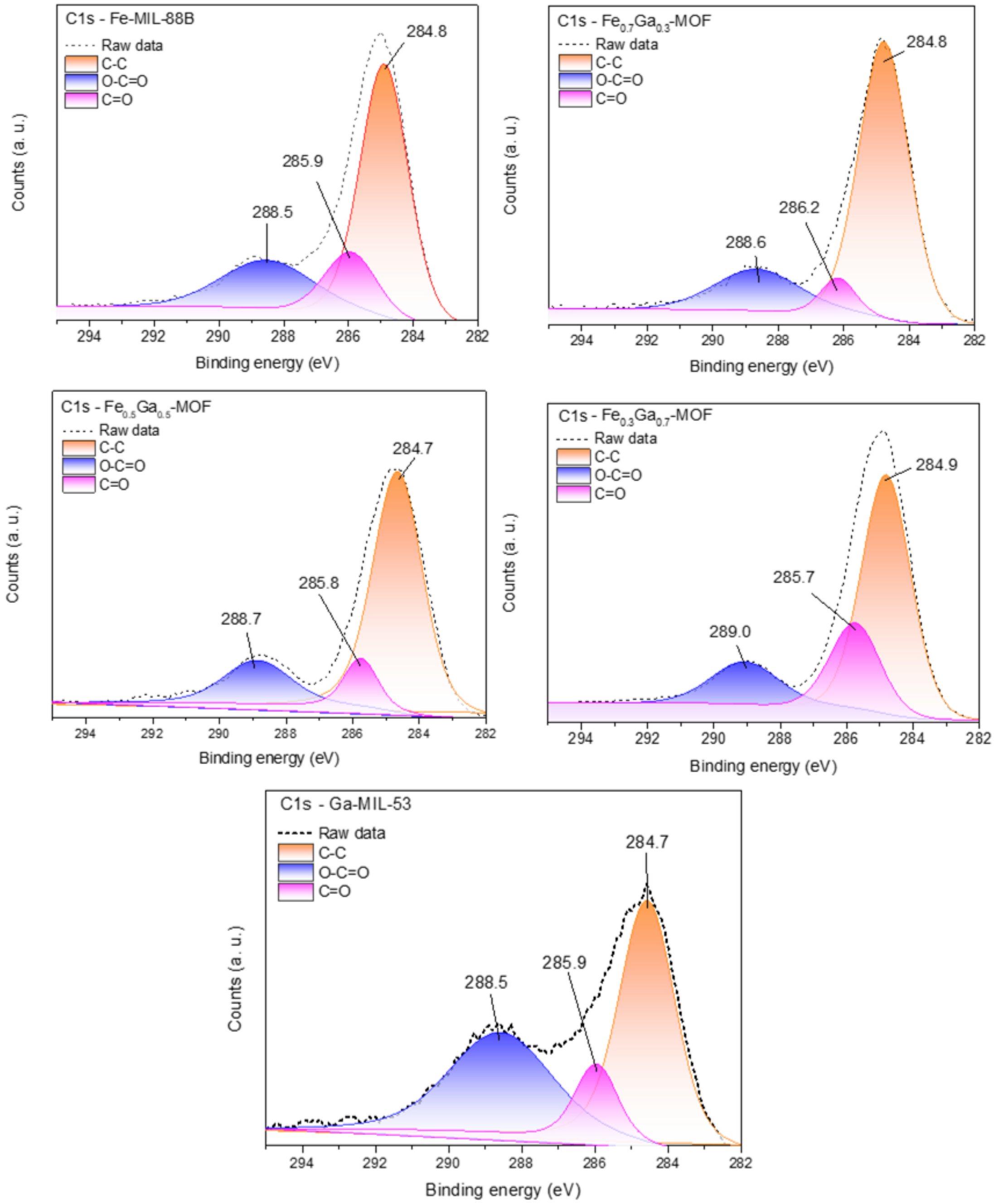

Figure S8. Comparison of carbon (C) high-resolution XPS spectra of each MOF.

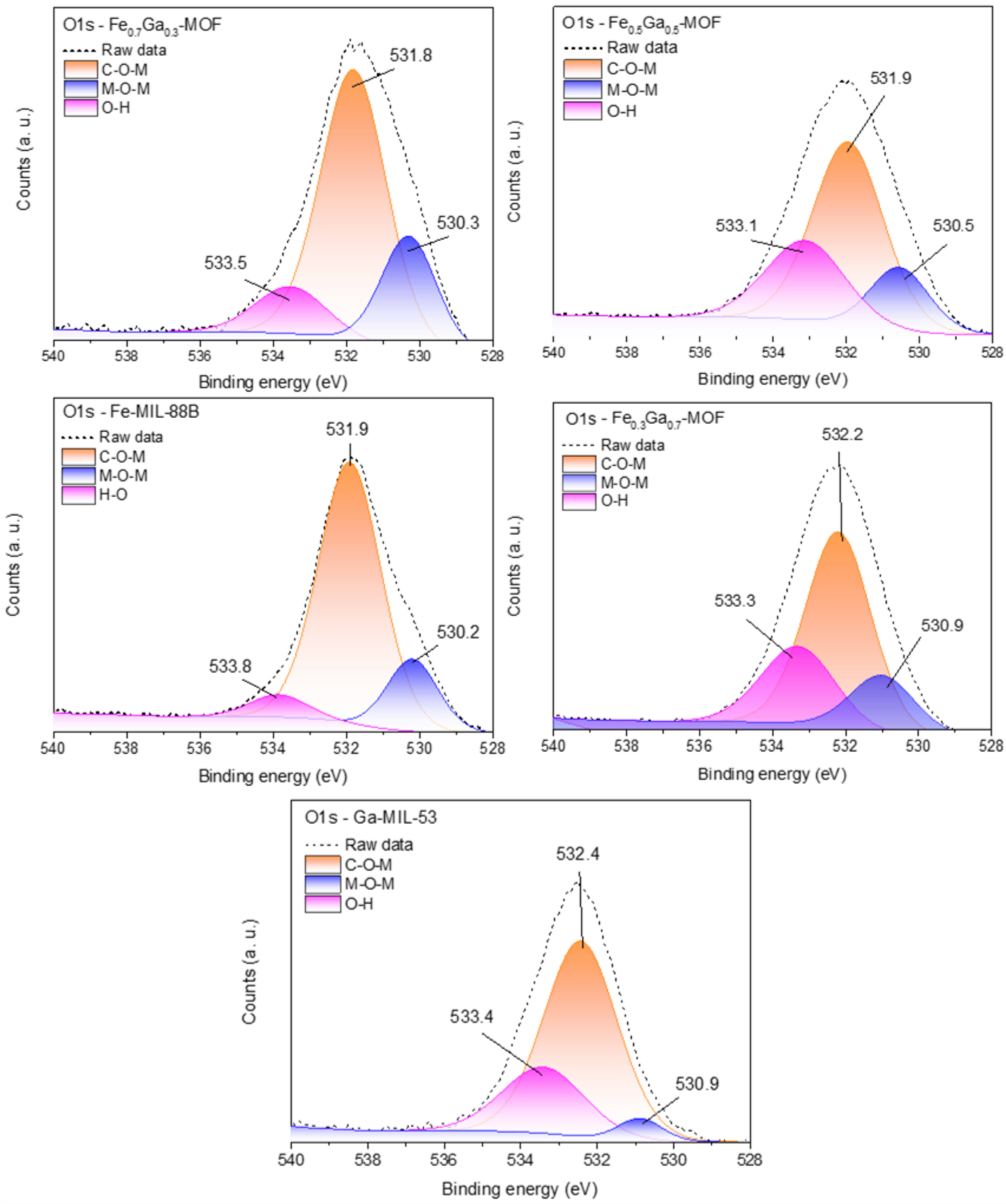

Figure S9. Comparison of oxygen (O) high-resolution XPS spectra of each MOF.

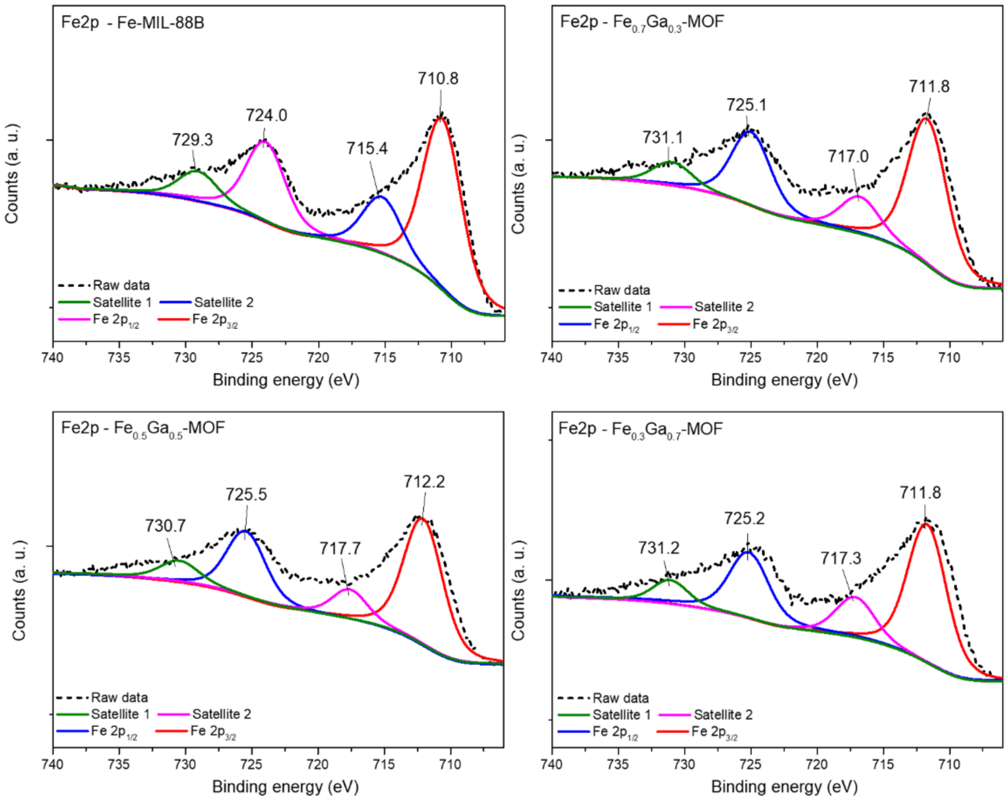

Figure S10. Comparison of iron (Fe) high-resolution XPS spectra of each MOF.

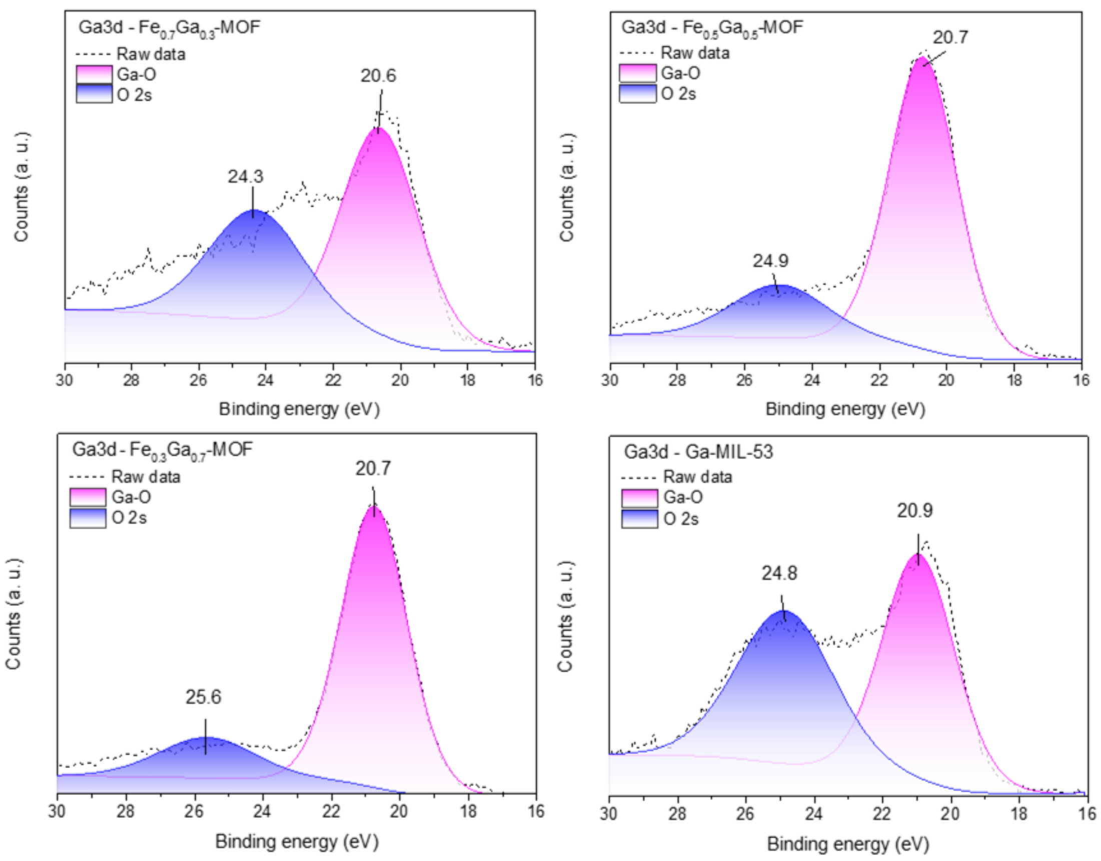

Figure S11. Comparison of gallium (Ga) high-resolution XPS spectra of each MOF.

Table S2. Binding energies of Fe2p and Ga3d XPS high-resolution spectra for the MOFs.

Sample	Binding energy (eV)					
	Fe^{3+} 2p$_{3/2}$	Fe^{3+} 2p$_{3/2}$ sat.	Fe^{3+} 2p$_{1/2}$	Fe^{3+} 2p$_{1/2}$ sat.	Ga-O	O 2s
Fe-MIL-88B	710.8	715.4	724.0	729.3	-	-
$Fe_{0.7}Ga_{0.3}$-MOF	711.8	717.0	725.1	731.1	20.6	24.3
$Fe_{0.5}Ga_{0.5}$-MOF	712.2	717.7	725.5	730.7	20.7	24.9
$Fe_{0.3}Ga_{0.7}$-MOF	711.8	717.3	725.2	731.2	20.7	25.6
Ga-MIL-53	-	-	-	-	20.9	24.8

Sat. = Satellite peak.

Table S3. Elemental composition of the catalysts synthesized.

Sample	Synthesis Molar Feeding Ratio (%)	Actual Molar Ratio (%)	Actual Weight Ratio (%)	Fe (%)[a]	Ga (%)[a]	Metal[b]	C (%)	H (%)	N (%)	O (%)[c]
	Fe:Ga	Fe:Ga	Fe:Ga							
Fe-MIL-88B	100:0	100:0	100:0	20.4	0	20.4	40.5	2.7	0.0	36.4
$Fe_{0.7}Ga_{0.3}$-MOF	70:30	79:21	75:25	18.1	5.9	24.1	38.3	2.2	0.3	33.8
$Fe_{0.5}Ga_{0.5}$-MOF	50:50	61:39	56:44	12.8	10.1	22.9	39.3	2.3	0.2	35.9
$Fe_{0.3}Ga_{0.7}$-MOF	30:70	41:59	36:64	8.6	15.3	23.9	38.6	2.4	0.3	35.0
Ga-MIL-53	0:100	0:100	0:100	0	25.2	25.2	38.2	2.4	0.2	34.1

[a] Fe and Ga (% weight in the solid). [b] Metal = sum of Fe and Ga. [c] Made by difference (O% = 100% – (Metal% + C% + N% + H%).

Table S4. Comparison of thermal decomposition from TGA.

Sample	Final temperature per stage / Remaining Weight (%)		
Fe-MIL-88B	200°C / 92.8%	300 °C / 80.7%	400 °C / 29.9%
$Fe_{0.7}Ga_{0.3}$-MOF	200°C / 99.8%	300 °C / 97.5%	425 °C / 37.5%
$Fe_{0.5}Ga_{0.5}$-MOF	200°C / 99.8%	300 °C / 97.6%	425 °C / 35.8%
$Fe_{0.3}Ga_{0.7}$-MOF	200°C / 99.8%	335 °C / 91.7%	425 °C / 35.7%
Ga-MIL-53	200°C / 99.7%	315 °C / 94.8%	555 °C / 36.1%

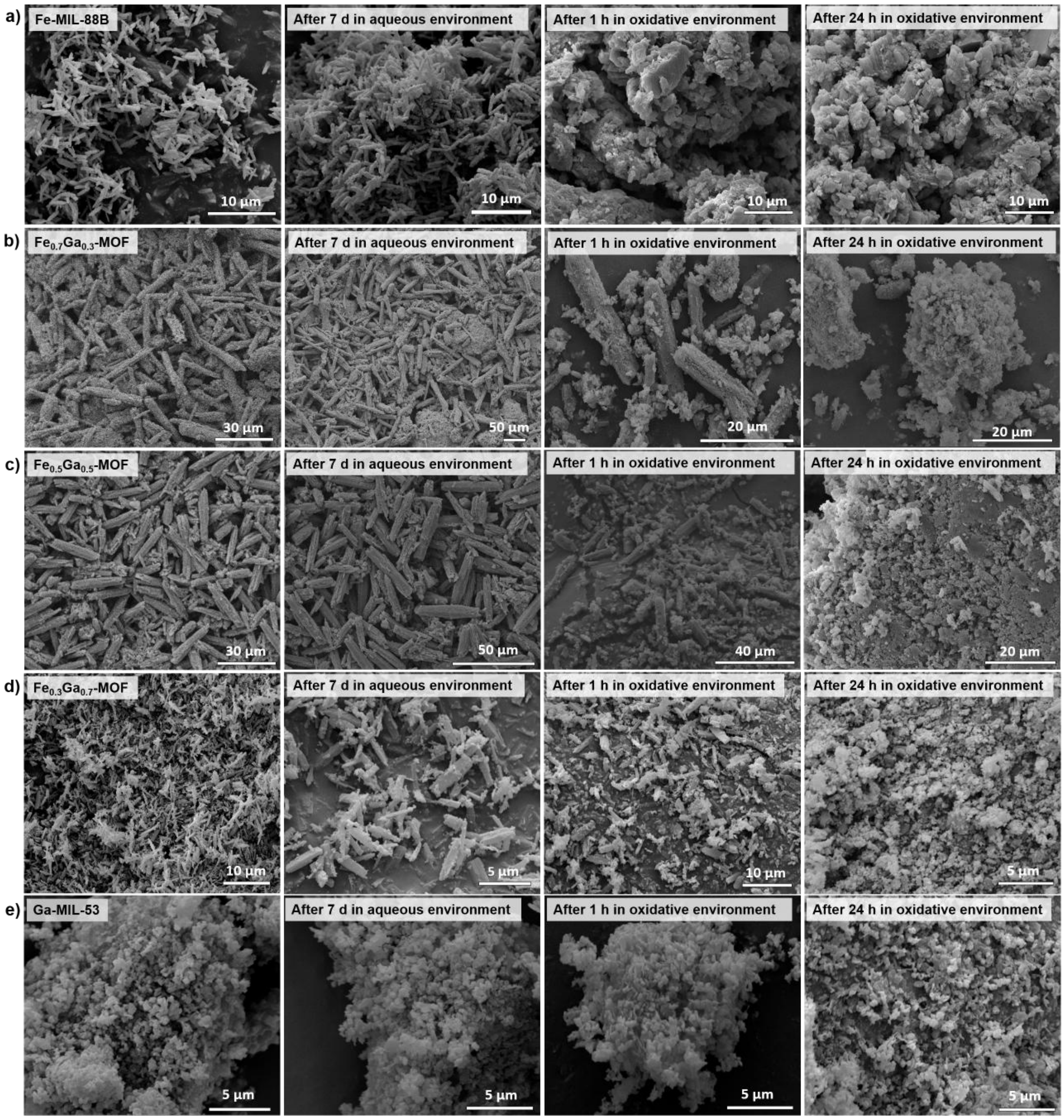

Figure S12. SEM of the MOFs stability tests in aqueous and oxidative environment. Each line is related to one MOF and the column represent it in a specific time after the stability test.

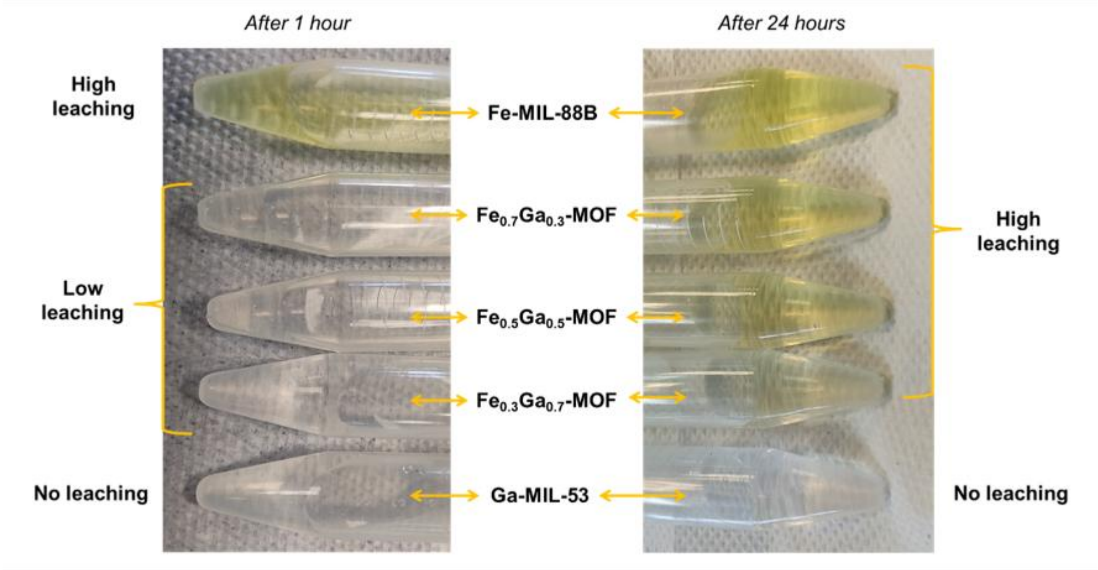

Figure S13. Comparison of color of the liquid phase from stability test in oxidative environment (1 h – left and 24 h - right).

Additional tests in Table S5 prove that all methanol comes from methane. Also, the H_2O_2 conversion is influenced by the catalysts and atmosphere.

Table S5. Summary of reaction conditions and results obtained for methane oxidation.

Entry	Sample	Time (h)	H_2O_2 conversion (%)	Productivity				
				Methanol (µmol)	Formic acid (µmol)	Acetic acid (µmol)	CO_2 (µmol)	Total (µmol)
**1**	Fe-MIL-88B	1	96	1.07	13.66	5.20	226.71	244.25
**2**	$Fe_{0.3}Ga_{0.7}$-MOF	1	31	0.59	9.74	2.10	12.79	22.83
**3**	$Fe_{0.3}Ga_{0.7}$-MOF	5	62	0.29	2.90	4.90	NQ	8.09
**4**	Ga-MIL-53	1	4	0	2.07	0.31	0	2.3
**5**	Blank (without CH_4)[a]	5	7	0	2.27	0.35	0	2.6
**6**	Blank (without MOF)	1	1	0	2.19	0.48	0	2.7
**7**	Blank (without MOF)	5	8	0	2.10	0.37	0	2.4

Reaction conditions: 2 mL H_2O, 20 mg MOF, 1.31 mmol H_2O_2, 40 ºC, 20.7 bar CH_4, 1000 rpm. NQ – Not quantified. [a] 20.7 bar Argon instead of CH_4 using $Fe_{0.3}Ga_{0.7}$-MOF.

Calculation of catalytic parameters:

$$Productivity\ (\mu mol) = moles\ of\ product\ formed$$

$$Productivity\ (\mu mol\ g_{cat}^{-1}) = \frac{sum\ of\ moles\ of\ MeOH,\ FA\ and\ AA\ formed}{mass\ of\ catalyst\ used}$$

$$CO_2\ productivity\ (\mu mol\ g_{cat}^{-1}) = \frac{moles\ of\ CO_2\ formed}{mass\ of\ catalyst\ used}$$

$$Distribution\ of\ oxygenated\ products\ (\%) = \left(\frac{moles\ of\ product\ formed}{sum\ of\ moles\ of\ MeOH,\ FA,\ and\ AA\ formed}\right) * 100$$

$$Selectivity\ of\ products\ (\%) = \left(\frac{moles\ of\ product\ formed}{sum\ of\ moles\ of\ MeOH,\ FA,\ AA\ and\ CO_2\ formed}\right) * 100$$

$$H_2O_2\ conversion\ (\%) = \left(\frac{moles\ of\ hydrogen\ peroxide\ after\ reaction}{moles\ of\ hydrogen\ peroxide\ before\ reaction}\right) * 100$$

It is important to note that the term 'oxygenated products' refers specifically to methanol, formic acid, and acetic acid in the liquid phase. CO_2 is not considered an oxygenated product in this context.

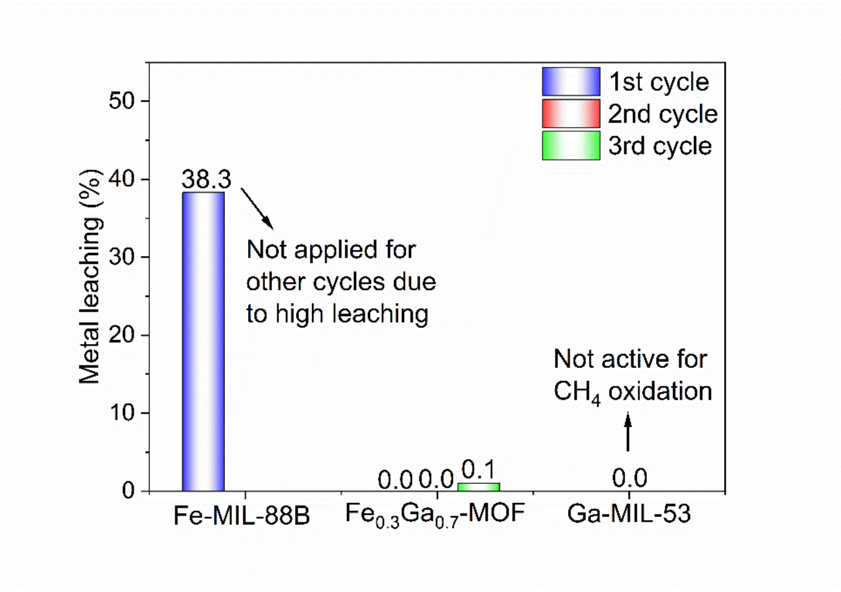

Figure S14. Evaluation of the **i**norganic composition of the materials after catalytic tests.

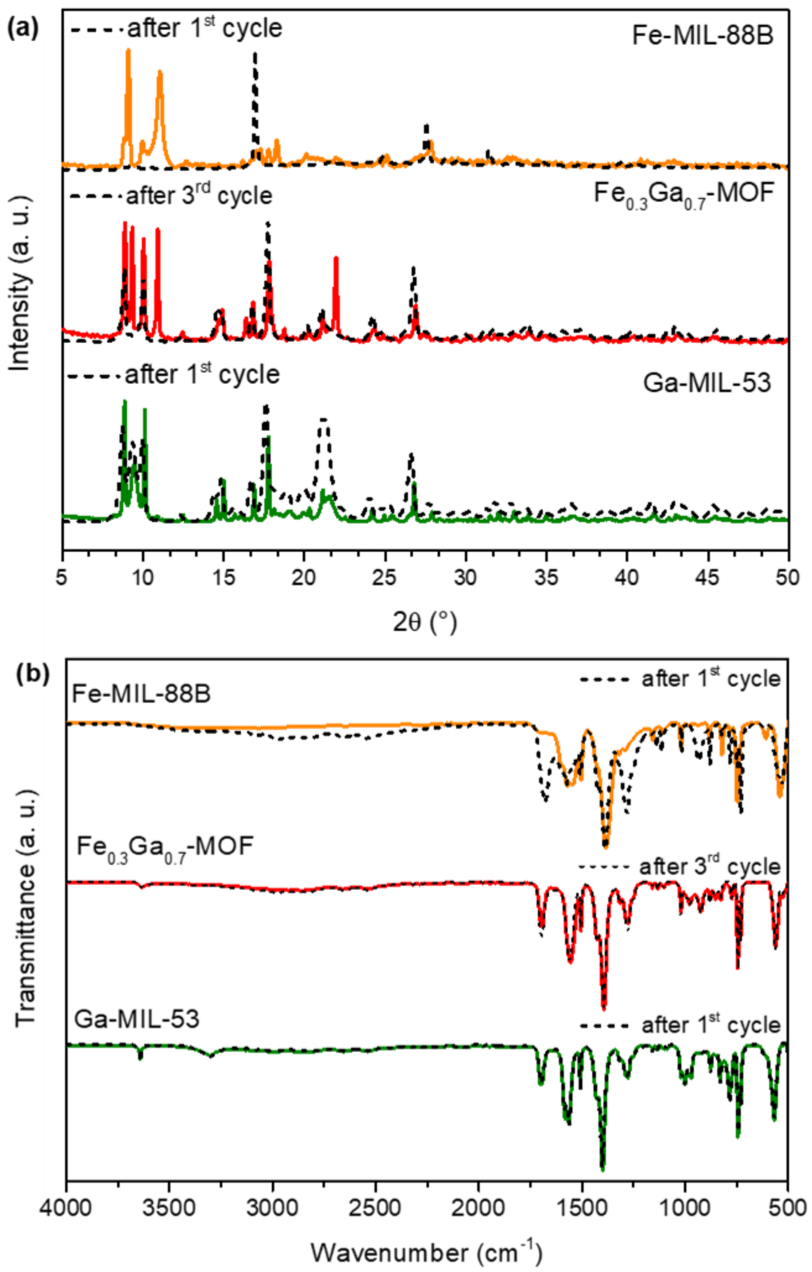

Figure S15. Comparison of **(a)** XRD and **(b)** FT-IR spectra of the MOFs materials before and after catalytic reactions. Straight line: pristine material. Dot line: after the reaction.

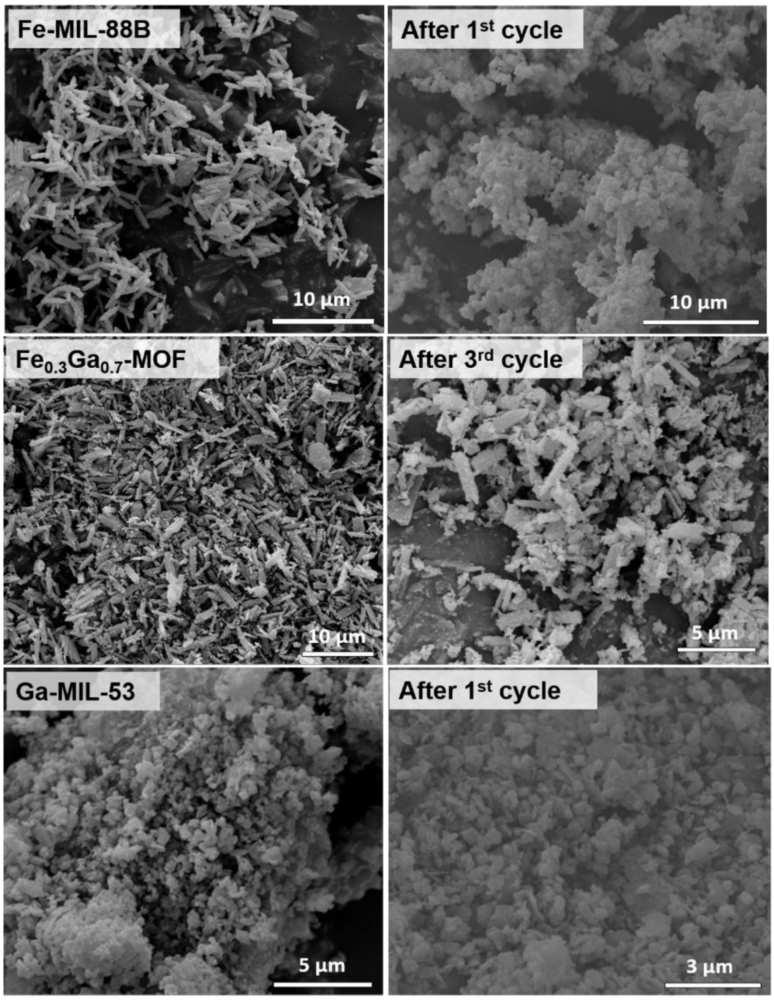

Figure S16. Comparison of the MOFs morphology before and after catalytic reactions by SEM.

Table S6. Comparison between the productivity of the direct methane oxidation to oxygenated products with different materials in the literature.

Catalyst	Oxidant	Experimental conditions	Productivities and distribution of oxygenates	Oxygenates & CO_2 total productivity and selectivity	Stability	Observations	Ref.
**$Fe_{0.3}Ga_{0.7}$-MOF**	H_2O_2 (1.31 mmol)	Batch: 40 °C, 20.7 bar CH_4, 1 h, 20 mg, 2 mL H_2O	MeOH (29.9 µmol g_{cat}^{-1}, 6%), FA (381.9 µmol g_{cat}^{-1}, 76%), and AA (90.1 µmol g_{cat}^{-1}, 18%)	Oxyg. (501.9 µmol $g_{cat}^{-}1$, 44%) and CO_2 (639.4 µmol g_{cat}^{-1}, 56%)	3 cycles (<10% activity loss)	One-step synthesis. Stable material.	This work
**Fe-MIL-88B (pristine)**	H_2O_2 (1.31 mmol)	Batch: 40 °C, 20.7 bar CH_4, 1 h, 20 mg, 2 mL H_2O	MeOH (53.5 µmol g_{cat}^{-1}, 6%), FA (578.3 µmol g_{cat}^{-1}, 66%), and AA (245.2 µmol g_{cat}^{-1}, 28%)	Oxyg. (877 µmol $g_{cat}^{-}1$, 7%) and CO_2 (11335 µmol g_{cat}^{-1}, 93%)	Not stable	Benchmark	This work
**MIL-53(Fe,Al)[a]**	H_2O_2 (5.06 mmol)	Batch: 60º C, 30 bar CH_4 4 h, 0.003 mol% of catalyst, 10 mL H2O [a]	MeOH (16 µmol, 51.6%), MHP (8 µmol, 25.8%), and FA (7 µmol, 22.6%)	Oxyg. (31 µmol, 77.5%) and CO_2 (9 µmol, 22.5%)	5 cycles (stable)	Harsher reaction conditions.	4
**Cu/ZIF-7**	H_2O_2 (5 mmol)	Batch: 50 °C, 30 bar CH_4, 4 h, 20 mg, and 10 mL H_2O	MeOH (177.4 µmol g_{cat}^{-1}, 29.0%), MHP (279.6 µmol g_{cat}^{-1}, 45.6%), and HMHP (155.7 µmol g_{cat}^{-1}, 25.4%)	Oxyg. (612.7 µmol g_{cat}^{-1}, 15%) and CO_2 (3498 µmol g_{cat}^{-1}, 85%)	5 cycles (<10% activity loss)	Multiple-step synthesis. High CO_2 prod. Harsher reaction conditions.	5
**UiO-66 (2.5TFA)-Fe**	H_2O_2 (2.94 mmol)	Batch: 50 °C, 30 bar CH_4, 1 h, 25 mg, and 9.7 mL D_2O,	MeOH (258.7 µmol g_{cat}^{-1}, 5.4%) MHP (401.0 µmol g_{cat}^{-1}, 8.4%), HMHP (1029.8 µmol g_{cat}^{-1}, 21.5%), and FA (3109 µmol g_{cat}^{-1}, 64.8%)	Oxyg. (4798.5 µmol $g_{cat}^{-}1$, 97.9%) and CO_2 (105 µmol g_{cat}^{-1}, 2.1%)	Not realized	Multiple-step synthesis. Harsher reaction conditions. No recycle data.	6
**Fe_2O_3**	H_2O_2 (2.94 mmol)	Batch: 50 °C, 30 bar CH_4, 1 h, 25 mg, and 9.7 mL D_2O,	MeOH (36.8 µmol g_{cat}^{-1}, 11.9%), MHP (59.2 µmol g_{cat}^{-1}, 22.9%), and HMHP (162.8 µmol g_{cat}^{-1}, 62.9%)	Oxyg. (258.8 µmol $g_{cat}^{-}1$, 73.8%) and CO_2 (92 µmol g_{cat}^{-1}, 26.2%)	Not realized	Benchmark. Lower oxyg. prod. No recycle data.	6
**FeO**	H_2O_2 (2.94 mmol)	Batch: 50 °C, 30 bar CH_4, 1 h, 25 mg, and 9.7 mL D_2O,	0 µmol	CO_2 (16 µmol g_{cat}^{-1}, 100%)	Not realized	Benchmark. No oxyg. prod. No recycle data.	6
**UiO-66-Fe**	H_2O_2 (2.94 mmol)	Batch: 50 °C, 30 bar CH_4, 1 h, 25 mg, and 9.7 mL D_2O,	MeOH (52.6 µmol g_{cat}^{-1}, 10.2%), MHP (60.5 µmol g_{cat}^{-1}, 11.7%), HMHP (35.3 µmol g_{cat}^{-1}, 6.8%), and FA (367.9 µmol g_{cat}^{-1}, 71.3%)	Oxyg. (516.3 µmol g_{cat}^{-1}, 95.9%) and CO_2 (22 µmol g_{cat}^{-1}, 4.1%)	Not realized	Multiple-step synthesis. Harsher reaction conditions	6

**$FeSO_4$ + 1%Pd/C[a]**	O_2 (10 bar)+ H_2 (3 bar)	Batch: 20° C, 15 bar CH_4, 0.5 h, [H_2SO_4] = 15mM, [Fe]=0.37mM, 50 mg, and 30 mL H_2O	MeOH (220 µmol g_{cat}^{-1}, 4.7%), FA (4360 µmol g_{cat}^{-1}, 92.4%), and MHP (140 µmol g_{cat}^{-1}, 3.0%)	Oxyg. (4720 µmol g_{cat}^{-1}, 65.6%) and CO_2 (2480 µmol g_{cat}^{-1}, 34.4%)	Not realized	Multiple-step synthesis. Use of strong acid. Noble metal. Homogeneous and heterogeneous species. No recycle data.	7
**Ce-UiO-Cu(OH)**	O_2 (6 bar)	Batch: 115 °C, 30 bar CH_4, 40 h, 3.7 mg, and 8 mL H_2O	AA (335100 µmol g_{cat}^{-1}, 100%)	Oxyg. (335100 µmol g_{cat}^{-1}, 96.0%), CO (1100 µmol g_{cat}^{-1}, 0.3%), and CO_2 (12700 µmol g_{cat}^{-1}, 3.6%)	3 cycles (stable)	Multiple-step synthesis. Noble metal. Harsher reaction conditions.	8
**UiO-67-Pt-Z**	H_2O_2 (48.95 mmol)	Batch: 60 °C, 50 bar CH_4, 2 h, 10 mg, and 5 mL of H_2O_2	MeOH (3%), EtOH (74.9%) and AA (21.9%)	Oxyg. (100%) and CO2 (0%)	Not realized	CO_2 was not quantified. Multiple-step synthesis. Harsher reaction conditions. No recycle data.	9
**H-ZSM-5**	O_2 (8 bar) and CO (10 bar)	Batch: 150 °C, 50 bar CH_4, 12 h, 28 mg, and 10 mL H_2O	MeOH (131.1 µmol g_{cat}^{-1}, 46.9%), FA (81.4 µmol g_{cat}^{-1}, 29.2%), and AA (66.8 µmol g_{cat}^{-1}, 23.9%)	ND	Not realized	Harsher reaction conditions. Lower oxyg. prod. No recycle data.	10
**0.5%wt Fe^{+3}/ZSM-5(30)COMM**	H_2O_2 (0.5 M)	Batch: 20 °C, 30.5 bar CH_4, 0.5 h, and 27 mg of catalyst	MeOH (740.7 µmol g_{cat}^{-1}, 23.9%), FA (2055.6 µmol g_{cat}^{-}1, 66.2%), and MHP (307 µmol g_{cat}^{-1}, 9.9%)	Oxyg. (3103.3 µmol g_{cat}^{-1}, 85.5%) and CO_2 (525.9 µmol g_{cat}^{-1}, 14.5%)	Not realized	Multiple-step synthesis. Harsher reaction conditions. No recycle data.	11
**Fe-silicate-1SYN**	H_2O_2 (0.5 M)	Batch: 20 °C, 30.5 bar CH_4, 0.5 h, and 27 mg of catalyst	MeOH (651.8 µmol g_{cat}^{-1}, 21.8%), FA (2040.7 µmol g_{cat}^{-}1, 66.6%), and MHP (370 µmol g_{cat}^{-1}, 12.2%)	Oxyg. (3062.5 µmol g_{cat}^{-1}, 92.8%) and CO_2 (237.0 µmol g_{cat}^{-1}, 7.2%)	5 cycles (<10% activity loss)	Multiple-step synthesis. Harsher reaction conditions.	11
**Au/ZSM-5**	O_2 (1 bar) and CO (1 bar)	Batch: 240 °C, 22.2 bar CH_4, 1 h, 100 mg, and 15 mL H_2O	MeOH (233.3 µmol g_{cat}^{-1}, 90.3%), AA (21.9 µmol g_{cat}^{-1}, 8.5), and PA (3.2 µmol g_{cat}^{-1}, 1.2%)	Oxyg. (258.4 µmol g_{cat}^{-1}, 16.7%) and CO_2 (1289.6 µmol g_{cat}^{-1}, 83.3%)	Not realized	Multiple-step synthesis. High CO_2 prod. Harsher reaction conditions.	12

MeOH = Methanol; FA = Formic acid; AA = Acetic acid; DME = Dimethyl ether; MHP = Methylhydroperoxide; HMHP = Hydroxymethyl hydroperoxide; EtOH = Ethanol; PA = Paracetic acid. Oxygenates are considered all the products obtained in the reaction excluding CO_2. The data from Fe-MIL-88B is due oxidation of CH_4 but accompanied of Fe leached species that may be active. Productivity means the quantity of the product per gram of catalyst used. Distribution of products means the percentage of each product based on the total productivity. ND = not determined or not enough data for calculating. [a] – The authors did not mention the exact mass of catalyst used.

References

(1) Correia, G. A.; de Araújo, M. L.; Carvalho, W. A.; Sairre, M.; Cerchiaro, G.; Shul'pina, L. S.; Kozlov, Y. N.; Shul´pin, G. B.; Kirillov, A. M.; Mandelli, D. Metal-Ligand Cooperation in the Catalytic Oxidation of (R)-Carvone by Ga(NO3)3/H2O2. *Molecular Catalysis* **2022**, *528* (June), 112457. https://doi.org/10.1016/j.mcat.2022.112457.

(2) Liu, J.; Ran, Z.; Cao, Q.; Ji, S. Preparation of MIL-88B(Fex,Co1−x) Catalysts and Their Application in One-Step Liquid-Phase Methanol Oxidation to Methyl Formate Using H2O2. *Chinese Journal of Catalysis* **2021**, *42* (12), 2254–2264. https://doi.org/10.1016/S1872-2067(20)63749-4.

(3) Volkringer, C.; Loiseau, T.; Guillou, N.; Férey, G.; Elkaïm, E.; Vimont, A. XRD and IR Structural Investigations of a Particular Breathing Effect in the MOF-Type Gallium Terephthalate MIL-53(Ga). *Journal of the Chemical Society. Dalton Transactions* **2009**, *53* (12), 2241–2249. https://doi.org/10.1039/b817563b.

(4) Osadchii, D. Y.; Olivos-Suarez, A. I.; Szécsényi, Á.; Li, G.; Nasalevich, M. A.; Dugulan, I. A.; Crespo, P. S.; Hensen, E. J. M.; Veber, S. L.; Fedin, M. V.; Sankar, G.; Pidko, E. A.; Gascon, J. Isolated Fe Sites in Metal Organic Frameworks Catalyze the Direct Conversion of Methane to Methanol. *ACS Catal* **2018**, *8* (6), 5542–5548. https://doi.org/10.1021/acscatal.8b00505.

(5) Lee, H.; Kwon, C.; Keum, C.; Kim, H.-E.; Lee, H.; Han, B.; Lee, S.-Y. Methane Partial Oxidation by Monomeric Cu Active Center Confined on ZIF-7. *Chemical Engineering Journal* **2022**, *450*, 138472. https://doi.org/10.1016/j.cej.2022.138472.

(6) Zhao, W.; Shi, Y.; Jiang, Y.; Zhang, X.; Long, C.; An, P.; Zhu, Y.; Shao, S.; Yan, Z.; Li, G.; Tang, Z. Fe-O Clusters Anchored on Nodes of Metal–Organic Frameworks for Direct Methane Oxidation. *Angewandte Chemie International Edition* **2021**, *60* (11), 5811–5815. https://doi.org/10.1002/anie.202013807.

(7) Kang, J.; Park, E. D. Aqueous-Phase Selective Oxidation of Methane with Oxygen over Iron Salts and Pd/C in the Presence of Hydrogen. *ChemCatChem* **2019**, *11* (17), 4247–4251. https://doi.org/10.1002/cctc.201900919.

(8) Antil, N.; Chauhan, M.; Akhtar, N.; Kalita, R.; Manna, K. Selective Methane Oxidation to Acetic Acid Using Molecular Oxygen over a Mono-Copper Hydroxyl Catalyst. *J Am Chem Soc* **2022**. https://doi.org/10.1021/jacs.2c12042.

(9) Xia, M.; Qiu, L.; Li, Y.; Shen, T.; Sui, Z.; Feng, L.; Chen, Q. A Metal-Organic Frameworks Composite Catalyst Containing Platinum and Polyoxometalate for Direct Conversion of Methane. *Mater Lett* **2022**, *307* (October 2021), 131078. https://doi.org/10.1016/j.matlet.2021.131078.

(10) Tang, Y.; Li, Y.; Fung, V.; Jiang, D. E.; Huang, W.; Zhang, S.; Iwasawa, Y.; Sakata, T.; Nguyen, L.; Zhang, X.; Frenkel, A. I.; Tao, F. Single Rhodium Atoms Anchored in Micropores for Efficient Transformation of Methane under Mild Conditions. *Nat Commun* **2018**, *9* (1), 1–11. https://doi.org/10.1038/s41467-018-03235-7.

(11) Hammond, C.; Dimitratos, N.; Jenkins, R. L.; Lopez-Sanchez, J. A.; Kondrat, S. A.; Hasbi Ab Rahim, M.; Forde, M. M.; Thetford, A.; Taylor, S. H.; Hagen, H.;

Stangland, E. E.; Kang, J. H.; Moulijn, J. M.; Willock, D. J.; Hutchings, G. J. Elucidation and Evolution of the Active Component within Cu/Fe/ZSM-5 for Catalytic Methane Oxidation: From Synthesis to Catalysis. *ACS Catal* **2013**, *3* (4), 689–699. https://doi.org/10.1021/cs3007999.

(12) Cao, J.; Lewis, R. J.; Qi, G.; Bethell, D.; Howard, M. J.; Harrison, B.; Yao, B.; He, Q.; Morgan, D. J.; Ni, F.; Sharma, P.; Kiely, C. J.; Li, X.; Deng, F.; Xu, J.; Hutchings, G. J. Methane Conversion to Methanol Using Au/ZSM-5 Is Promoted by Carbon. *ACS Catal* **2023**, *13* (11), 7199–7209. https://doi.org/10.1021/acscatal.3c01226.
